# Reconstructing single-cell karyotype alterations in colorectal cancer identifies punctuated and gradual diversification patterns

**DOI:** 10.1038/s41588-021-00891-2

**Published:** 2021-07-01

**Authors:** Yannik Bollen, Ellen Stelloo, Petra van Leenen, Myrna van den Bos, Bas Ponsioen, Bingxin Lu, Markus J. van Roosmalen, Ana C. F. Bolhaqueiro, Christopher Kimberley, Maximilian Mossner, William C. H. Cross, Nicolle J. M. Besselink, Bastiaan van der Roest, Sander Boymans, Koen C. Oost, Sippe G. de Vries, Holger Rehmann, Edwin Cuppen, Susanne M. A. Lens, Geert J. P. L. Kops, Wigard P. Kloosterman, Leon W. M. M. Terstappen, Chris P. Barnes, Andrea Sottoriva, Trevor A. Graham, Hugo J. G. Snippert

**Affiliations:** 1 Molecular Cancer Research, Center for Molecular Medicine, University Medical Center Utrecht, Utrecht University, Utrecht, the Netherlands; 2 Oncode Institute, Utrecht, the Netherlands; 3 Medical Cell Biophysics, TechMed Centre, University of Twente, Enschede, the Netherlands; 4 Department of Genetics, Center for Molecular Medicine, University Medical Center Utrecht, Utrecht University, Utrecht, the Netherlands; 5 Department of Cell and Developmental Biology, University College London, London, UK; 6 UCL Genetics Institute, University College London, London, UK; 7 Hubrecht Institute, KNAW, Utrecht, the Netherlands; 8 University Medical Center Utrecht, Utrecht, the Netherlands; 9 Centre for Genomics and Computational Biology, Barts Cancer Institute, Barts and the London School of Medicine and Dentistry, Queen Mary University of London, London, UK; 10 UCL Cancer Institute, UCL, London, UK; 11 Hartwig Medical Foundation, Amsterdam, the Netherlands; 12 Evolutionary Genomics and Modelling Lab, Centre for Evolution and Cancer, The Institute of Cancer Research, London, UK

**Keywords:** Colorectal cancer, DNA sequencing, Time-lapse imaging

## Abstract

Central to tumor evolution is the generation of genetic diversity. However, the extent and patterns by which de novo karyotype alterations emerge and propagate within human tumors are not well understood, especially at single-cell resolution. Here, we present 3D Live-Seq—a protocol that integrates live-cell imaging of tumor organoid outgrowth and whole-genome sequencing of each imaged cell to reconstruct evolving tumor cell karyotypes across consecutive cell generations. Using patient-derived colorectal cancer organoids and fresh tumor biopsies, we demonstrate that karyotype alterations of varying complexity are prevalent and can arise within a few cell generations. Sub-chromosomal acentric fragments were prone to replication and collective missegregation across consecutive cell divisions. In contrast, gross genome-wide karyotype alterations were generated in a single erroneous cell division, providing support that aneuploid tumor genomes can evolve via punctuated evolution. Mapping the temporal dynamics and patterns of karyotype diversification in cancer enables reconstructions of evolutionary paths to malignant fitness.

## Abstract (web summary)

Analysis of live-cell imaging and single-cell genome sequencing data of colorectal cancer organoids identifies temporal dynamics of sub-chromosomal copy-number amplifications.

## Main

Aneuploidy—defined as a cell having an abnormal number of chromosomes or sub-chromosomal fragments—is among the most common features of human cancers^1^. The prevalence of aneuploidy and the detection of recurrent copy-number alterations (CNAs) across cancer types indicate that genome-wide aneuploidy plays an active role in promoting malignant phenotypes^2,3^.

How an aneuploid tumor genome evolves over time remains incompletely resolved. Although single-cell sequencing from patient samples captures genetic diversity in human tumors^4,5^, the evolutionary history of an aneuploid genome is difficult to determine, as the two critical parameters that fuel its progression—ongoing chromosomal instability (CIN) and selection pressures—are dynamically intertwined^3,6–8^. Consequently, general models of tumor evolution are still under debate, in part because the karyotype alteration rate and the patterns by which de novo karyotypes emerge and propagate within human tumors are not well understood^6,8,9^. These parameters in particular are difficult to extract from patient material since it provides limited to no information on genetic intermediates and the timescales that separate individual tumor subclones (the number of cell generations). For instance, punctuated bursts of karyotype alterations in human cancers have been described^10–13^, but the exact timescale and prevalence of such events remain elusive^14^.

Patient-derived tumor organoids (PDTOs) offer a unique opportunity to address these fundamental questions. PDTOs are three-dimensional (3D) mini-organs derived from primary tumor tissue. PDTOs maintain the histopathological features of the native tumor, including high concordance in somatic mutations and transcriptome and drug response profiles^15–17^. Crucially, PDTO models are the closest representatives of human tumors that are compatible with high-spatiotemporal-resolution imaging of cells, enabling live microscopy of clonal tumor outgrowth.

## Results

### De novo CNAs emerge and propagate during PDTO outgrowth

**Fig. 1:**
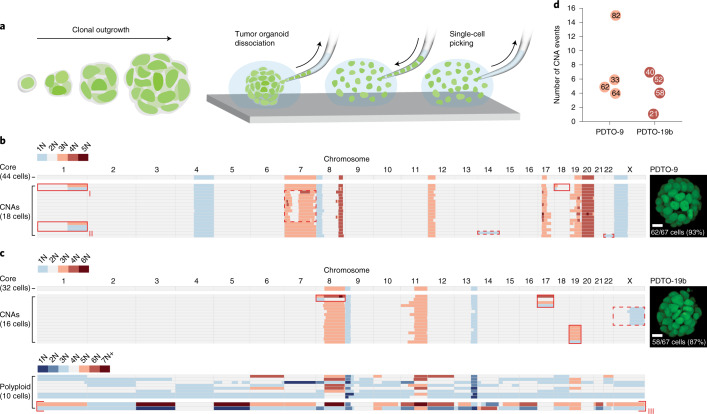
Chromosomal CNAs emerge and propagate during clonal PDTO outgrowth. **a**, Schematic of the procedure to perform single-cell whole-genome sequencing of the entire cell population from individual organoids. Clonal PDTO structures are mechanically dissociated into single cells before manual picking of individual cells for prospective genome-wide CNA analysis. **b**, Karyotype heatmap showing 62 cells derived from a clonal PDTO-9 structure expressing transgenic H2B-Dendra2 and consisting of 67 cells (93% recovery). A total of 18 cells showed de novo CNAs. Reciprocal gains and losses are indicated with solid red boxes. Dashed boxes indicate CNA events where a reciprocal loss or gain is missing. Sub-chromosomal CNAs were counted as events when represented in more than one cell. Parallel emergence of de novo CNAs involving chromosome 1q (lineages I and II) was determined by co-occurrence of a sub-chromosomal CNA in chromosome 18. Scale bar, 10 μm. **c**, As in **b**. The sequencing data of 58 PDTO-19b cells are shown (87% recovery). A total of 16 cells showed de novo CNAs. The bottom panel shows a population of polyploid cells with large deviations from the core karyotype. Two cells show reciprocal gains and losses across their genome (lineage III). Scale bar, 10 μm. **d**, Graph indicating the number of CNA events per dataset (eight datasets). The dataset size is indicated within each circle. Events include reciprocal CNAs, non-reciprocal whole-chromosome gains or losses and non-reciprocal sub-chromosomal CNAs represented in more than one cell. Hopeful monster karyotypes were excluded from this analysis. CNAs that occurred in the same cell division were considered as one event.

**Extended Data Fig. 1:**
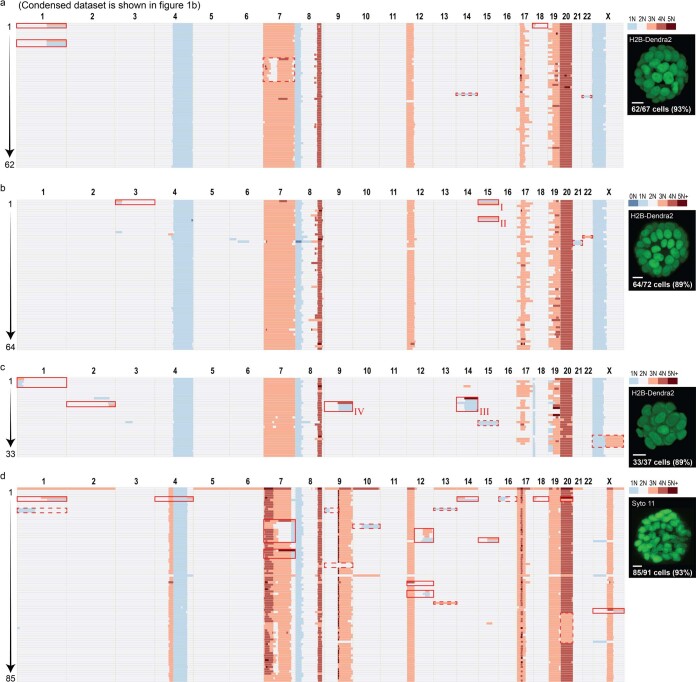
Full and additional whole population PDTO sequencing datasets of PDTO-9. **a**, Full PDTO-9 dataset of experiment shown in Fig. 1b. Karyotype heatmap showing 62 cells derived from a clonal PDTO-9 structure expressing transgenic H2B-Dendra2 and consisting of 67 cells (93% recovery). Reciprocal gains and losses are indicated with red boxes. Dashed boxes indicate CNA events where a reciprocal loss or gain is missing. Sub-chromosomal CNAs were counted as events when represented in more than one cell. Scalebar is 10μm. **b**, As in a. 64 out of 72 cells provided quality sequence (89% recovery). Parallel emergence of de novo whole-chromosome missegregations of Chr.15 (I, II) was determined by co-occurrence of a sub-chromosomal CNA in Chr.3. **c**, As in a. 33 out of 37 cells provided quality sequence (89% recovery). Two lineages show amplified sub-chromosomal CNAs in one cell, while all other cells in the lineage show a reciprocal loss of the same region (III. IV). Consistent with replication and collective missegregation of acentric chromosomal fragments. See Fig. 3. **d**, As in a. Genetically unmodified PDTO-9 organoid. Cells were stained with Syto 11 to allow single-cell picking. 85 out of 91 cells provided quality sequence (93% recovery).

**Extended Data Fig. 2:**
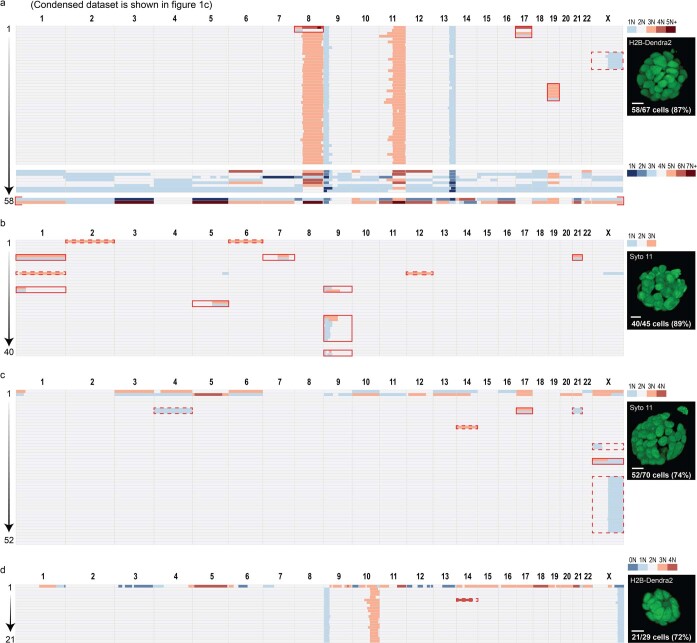
Full and additional whole population PDTO sequencing datasets of PDTO-19b. **a**, Full PDTO-19b dataset of experiment shown in Fig. 1c. Karyotype heatmap showing 58 cells derived from a clonal PDTO-19b structure expressing transgenic H2B-Dendra2 and consisting of 67 cells (87% recovery). Reciprocal gains and losses are indicated with red boxes. Dashed boxes indicate CNA events where a reciprocal loss or gain is missing. Sub-chromosomal CNAs were counted as events when represented in more than one cell. The bottom panel shows a population of polyploid cells with large deviations from the core karyotype (hopeful monsters). Two cells show reciprocal gains and losses across their genome (red brackets). Scalebar is 10μm. **b**, As in a. Genetically unmodified PDTO-19b organoid. Cells were stained with Syto 11 to allow single cell picking. 40 out of 45 cells provided quality sequence (89% recovery). **c**, As in a. Genetically unmodified PDTO-19b organoid. Cells were stained with Syto 11 to allow single cell picking. 52 out of 70 cells provided quality sequence (74% recovery). **d**, As in a. 21 out of 29 cells were sequenced (72% recovery). The previously reported TP53 mutation (chr 17: 7577121 G > A) for PDTO-19b [van de Wetering, 2016] was confirmed in all sequenced clonal organoids.

**Extended Data Fig. 3:**
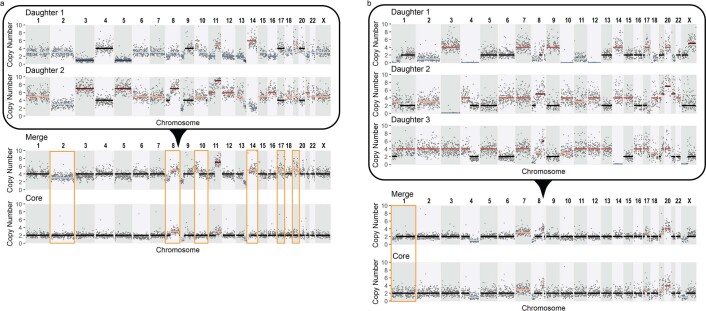
Copy-number profiles of hopeful monster karyotypes. **a**, Reciprocal copy-number profiles of two PDTO-19b cells with hopeful monster karyotypes are shown (Fig. 1c, III). Since the ancestor karyotype of these two cells is unclear, reciprocity between hopeful monster karyotypes refers to the observation that all chromosomal segments add up to an even number. Grey dots indicate bin ratios (Bin size: 1 Mbp). Integer copy-number states are indicated with black (no deviation of a tetraploid state), blue (loss) and orange (gain) lines. Below: The copy-number profile of the merged sequencing data of the two hopeful monsters mimics a duplicated core karyotype of the sequenced PDTO-19b organoid. Orange boxes indicate CNAs in the merged profile that deviate from a duplication of the core karyotype, indicating that these CNAs were present in the ancestor cell. **b**, Reciprocal copy-number profiles of three PDTO-9 cells with hopeful monster karyotypes are shown (Fig. 4a). Grey dots indicate bin ratios (Bin size: 1 Mbp). Integer copy-number states are indicated with black (no deviation of a tetraploid state), blue (loss) and orange (gain) lines. Adding up the copy-number state of chromosome segments across all three hopeful monster karyotypes results in a twice replicated genome (‘ploidy 8’, not shown). Below: The copy-number profile of the merged sequencing data mimics the core karyotype carrying a de novo loss of 1pter-p34.2 (orange box).

To investigate the extent to which de novo chromosomal CNAs emerge and propagate during clonal outgrowth of a single tumor cell, we developed a protocol that allows single-cell whole-genome sequencing of the entire cell population derived from a single organoid (Fig. 1a). Using this protocol, we repeatedly sequenced a high fraction (~90%) of cells from clonal tumor organoids of two patients with colorectal cancer (CRC): PDTO-9 (microsatellite stable) and PDTO-19b (microsatellite unstable)^15^. Sequencing data from both PDTO lines displayed de novo whole-chromosome as well as sub-chromosomal CNAs among cells (Fig. 1b,c and Extended Data Figs. 1 and 2), in agreement with our previous study showing that both PDTOs are in CIN^7^. Capturing the entire population of cells from individual clonal PDTOs has the critical advantage of detecting reciprocal copy-number gains and losses between cells, indicating that the two lineages are progeny of the same ancestral CNA event (Fig. 1b,c, red boxes; >1 cell with the same CNA indicates propagation). Across datasets, CNA events occurred at a rate of approximately one in ten cell divisions for both PDTO-9 and PDTO-19b (Fig. 1d). Notably, we identified instances where identical CNAs emerged from independent events within a single PDTO-9 organoid, which were discriminated by the co-occurrence of additional CNAs that prohibited the assembly of a coherent single phylogeny (Fig. 1b (lineages I and II) and Extended Data Fig. 1b (lineages I and II)). Indeed, we frequently detected cell divisions where multiple CNAs arose simultaneously. Extreme cases were represented by a subset of PDTO-19b cells with highly aberrant karyotypes that could be classified as hopeful monsters^18,19^, defined as a genome-wide set of changes in ploidy that are likely to substantially alter fitness. Two of these cells displayed extensive reciprocal CNAs across their genomes (Fig. 1c, lineage III), indicating that they were the progeny of a single catastrophic cell division. The sum of both karyotypes pointed to a genome-duplicated ancestor, in line with models suggesting that extreme aneuploid states often evolve from unstable tetraploid intermediates^20^ (Extended Data Fig. 3a).

Collectively, these data indicate that de novo CNAs readily arise and propagate during PDTO outgrowth. Furthermore, we detected several instances of punctuated karyotype alterations that involved multiple chromosomes in a single-cell division, with hopeful monsters representing the most extreme cases.

### Integrating live-cell imaging and single-cell sequencing data using 3D Live-Seq

To further investigate the dynamics of de novo CNAs as they arise and propagate across cell generations, we developed 3D Live-Seq—a protocol inspired by the LookSeq strategy^21^. 3D Live-Seq incorporates confocal live-cell imaging data of a growing PDTO structure and single-cell sequencing data of the entire imaged cell population, to allow a precise reconstruction of evolving tumor karyotypes across consecutive cell generations. The expression of transgenic H2B-Dendra2 (ref. ^22^) is sufficiently bright to support long-term confocal imaging with remarkable spatiotemporal resolution. Importantly, cells of interest can be photoconverted from green to red Dendra2 fluorescence to serve as a reference landmark for the direct integration of imaging and sequencing datasets.

### Chromatin errors during mitosis are phenotypes of de novo CNAs

**Fig. 2:**
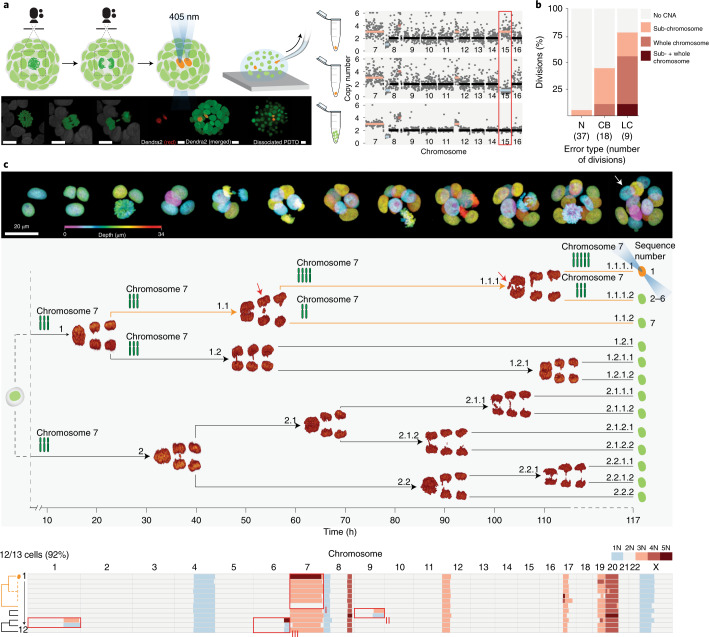
Capturing karyotype diversification across consecutive cell generations using 3D Live-Seq. **a**, Top: schematic representing confocal imaging of individual PDTO-9 organoids expressing H2B-Dendra (green) to capture high-spatiotemporal-resolution recordings of individual cell divisions, followed by photoconversion of cells of interest to red fluorescence before single-cell isolation. Bottom: representative imaging stills of the procedure. Right: CNA plots from chromosome 7 to chromosome 16 are shown for photoconverted daughter cells 1 and 2 (top two lanes), as well as the remaining single cells of the organoid combined in lane 3 as reference material. The red box indicates the reciprocal gain and loss of chromosome 15, matching the lagging chromatin phenotype. The dots represent measured CNAs per bin (1 Mb). The average deviation from the diploid genome is indicated by color coding (blue = loss; orange = gain). Scale bars, 10 μm. **b**, Bar graph representing the fraction of PDTO-9 cell divisions that resulted in reciprocal whole-chromosome or sub-chromosomal CNAs among daughter cells per chromatin error class. In total, 37 normal (N) divisions, 18 divisions displaying chromatin bridges (CB) and nine divisions with lagging chromatin (LC) were captured. **c**, 3D Live-Seq dataset of a PDTO-9 organoid consisting of 13 cells. Top: representative stills of the growing PDTO-9 structure, with nuclei coded in false color by depth. Middle: reconstruction of the true mitotic tree containing representative stills of 3D-rendered anaphases. The onset of anaphase is indicated by arrowheads in relation to the time axis. Bottom: karyotype heatmap of 12 cells (93% recovery) isolated from the imaged PDTO-9 organoid. A consecutive missegregation of chromosome 7 (lineage I) was mapped to the highlighted branch of the mitotic tree using the photoconverted cell (white arrow in top panel) as a reference landmark. Anaphase stills showing lagging chromatin are indicated with red arrows. Chromosome cartoons along the mitotic tree indicate copy-number changes across cell generations. Lineages II and III cannot be accurately mapped onto the mitotic tree.

**Extended Data Fig. 4:**
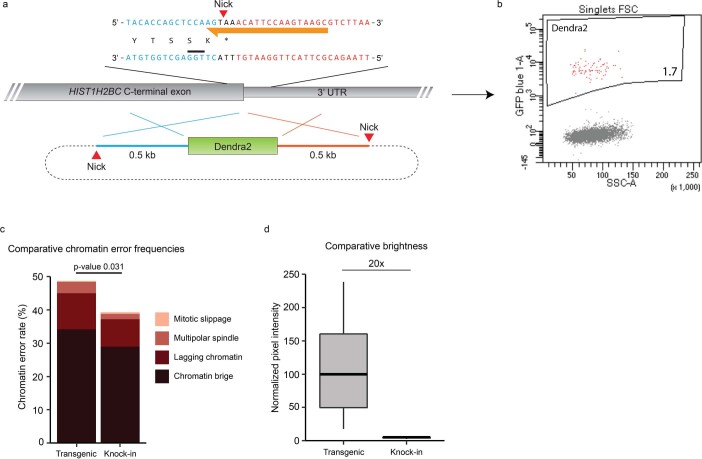
Transgenic H2B expression does not exacerbate CIN. **a**, The *HIST1H2BC* locus, coding for most abundant Histone H2B, was targeted to fluorescently tag H2B1C with Dendra2. Cas9 nickase (D10A) was targeted to the endogenous STOP codon, as well as both sites of the homology arms flanking the donor template. **b**, 18 days post electroporation, H2B-Dendra2 positive cells were isolated using FACS. Overall knock-in efficiency of 1.7% (not corrected for electroporation efficiency of ~10%). **c**, Similar CIN phenotypes of PDTO-9 expressing transgenic H2B-Dendra2 or carrying a *HIST1H2BC*-Dendra2 knock-in. 649 divisions across 34 organoids and 183 divisions across 27 organoids were scored from the transgenic and knock-in line respectively. Difference in scored chromatin error rates is statistically significant (Chi square test, p = 0.031), although the error rate in the knock-in line is likely underreported due to the lower brightness of endogenous H2B-Dendra2 expression. **d**, Fluorescent signal of H2B-Dendra was compared between PDTO-9 lines expressing transgenic H2B-Dendra2 (n = 95 cells, 19 organoids) or carrying a *HIST1H2BC*-Dendra2 knock-in (n = 100 cells, 20 organoids). Data are represented as box-and-whisker plots; boxes represent quartiles 2 and 3, the horizontal line represents the mean and whiskers represent minima and maxima within the 1.5× interquartile range.

We first implemented 3D Live-Seq to investigate whether de novo CNAs correlate with specific chromatin errors during mitosis. Collectively, we generated high-resolution imaging data of 64 cell divisions, each from individual tumor organoids (PDTO-9), to score their chromatin phenotype. We then sequenced both daughter cells along with a bulk sample consisting of the remaining cells of the dissociated PDTO structure (Fig. 2a). As expected, de novo CNAs were almost exclusively generated among daughter cells of cell divisions that involved either a chromatin bridge or lagging chromatin (Fig. 2b and Supplementary Note 1). Furthermore, chromatin bridges generally resulted in sub-chromosomal CNAs, whereas whole-chromosome missegregations were mainly associated with lagging chromatin. This bias was consistent with reports showing that chromatin bridges primarily represent the stretching of fused or dicentric chromosomes^23^, likely to result in sub-chromosomal fragments when broken. To exclude that the expression of transgenic H2B substantially exacerbates CIN phenotypes, we generated a CRISPR knock-in^24^ of Dendra2 at the carboxy terminus of the *HIST1H2BC* gene (Extended Data Fig. 4a,b). In a direct comparison, both lines showed clear CIN phenotypes with similar error-type frequency distributions (Extended Data Fig. 4c). The lower overall CIN rate in the knock-in line could be attributed to the substantially reduced brightness of the knock-in, which hampers effective scoring of subtle chromatin errors, as well as to clonal differences between lines^7^ (Extended Data Fig. 4d). Furthermore, single cells isolated from genetically unmodified PDTOs (using the DNA dye Syto 11) that had not been exposed to live-cell imaging procedures, displayed a similar number of de novo CNAs compared with other PDTO datasets (Extended Data Figs. 1d and 2b,c).

While these data confirm that chromatin errors are distinct phenotypes of karyotype diversification, chromatin bridges in particular often failed to generate detectable CNAs (Fig. 2b). In contrast with artificially induced chromatin bridges^25^, naturally occurring chromatin bridges in PDTOs are likely to reflect a more complete spectrum of underlying causalities, of which a subset may be resolved at high fidelity, while other cases may give rise to mutations that are below our detection threshold (Supplementary Note 2).

### 3D Live-Seq captures karyotype alterations across cell generations

Next, we applied 3D Live-Seq to study evolving tumor genomes across multiple consecutive cell divisions. To showcase the feasibility of our protocol, we recorded the unperturbed outgrowth of an individual PDTO-9 organoid across three cell generations (from two to 13 cells) and reconstructed the true mitotic tree with a detailed characterization of mitotic fidelity for each cell division (Fig. 2c). Before single-cell isolation, we selected one cell of interest for photoconversion based on a lagging chromatin phenotype during its preceding cell division (Fig. 2c, cell number 1.1.1.1). Single-cell sequencing data from 12 out of 13 cells displayed several de novo CNAs across three lineages (Fig. 2c, lineages I–III). Using the sequencing result of the photoconverted cell as a landmark and the mitotic tree as a structural constraint on potential phylogenetic solutions of lineage I, we mapped a consecutive missegregation of chromosome 7 to the highlighted branch within the mitotic tree (Fig. 2c, branch 1.1). In agreement with our previous data, divisions at which chromosome 7 missegregated displayed a lagging chromatin phenotype migrating toward the daughter cell that gained a copy of chromosome 7 (Fig. 2c, red arrows). The remaining de novo CNAs could not be mapped with high accuracy as their potential phylogenetic solutions matched multiple branches of the mitotic tree (Fig. 2c, lineages II and III). Lineage III displayed an unusual CNA of chromosome 6q (chromosome 6q24.2-ter) with one cell carrying five copies of chromosome 6q while its sister cell, identified by the reciprocal CNA of chromosome 1q, displayed only one copy of chromosome 6q. Given the structural constraints set forth by the mitotic tree, any conventional phylogenetic interpretation that could explain the origin of additional chromosome 6q copies appeared unlikely.

### Replication and collective missegregation of acentric chromosomal fragments

**Fig. 3:**
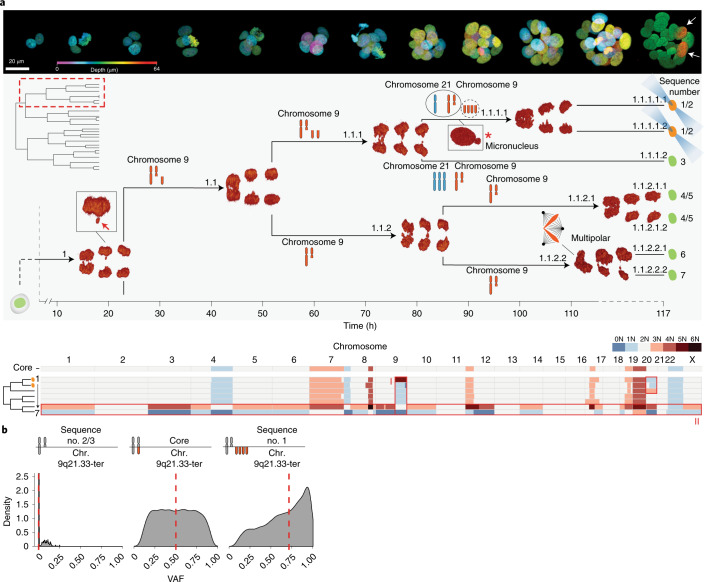
Acentric chromosomal fragments are prone to cycles of replication and collective missegregation. **a**, 3D Live-Seq dataset of a PDTO-9 organoid consisting of 25 cells (100% recovery). Top: representative stills of the growing PDTO-9 structure with nuclei coded in false color by depth. The final still shows two photoconverted cells (white arrows) that were the progeny of branch 1.1 of the mitotic tree. Branch 1.1 is highlighted (dashed red box) in the full tree structure in the top left of the middle panel and enlarged as the main image in the middle panel, with 3D-rendered stills of each anaphase. The onset of anaphase is indicated by arrowheads in relation to the time axis. Bottom: karyotype heatmap of all cells mapped to branch 1.1 (cells 1–7) and a reference core karyotype of the imaged PDTO. The sequence numbers of the mapped sequencing results in relation to the mitotic tree are indicated, including the photoconversion state of each cell. Chromosome cartoons along enlarged branch 1.1 indicate reconstructed copy-number changes across cell generations. The inset of cell division 1 shows a lagging chromatin structure, as indicated by the red arrow. The asterisk highlights the presence of a micronucleus in cell 1.1.1.1 that persists until nuclear envelope breakdown. **b**, The variant allele frequency (VAF) distribution of variants located on the acentric chromosome 9q fragment (Chr. 9q21.33-ter) is consistent with two rounds of replication and collective missegregation for cell number 1. The VAF of SNVs located on the missegregated chromosome 9q21.33-ter region is 0% for cell numbers 2 and 3 (only one copy of chromosome 9q) and 50% for a cell with the core karyotype. Cell number 1 shows nearly 80% VAF for these SNVs, indicative that four of the five chromosome 9q21.33-ter copies originated from the same parental allele. The dashed red line indicates the median.

**Extended Data Fig. 5:**
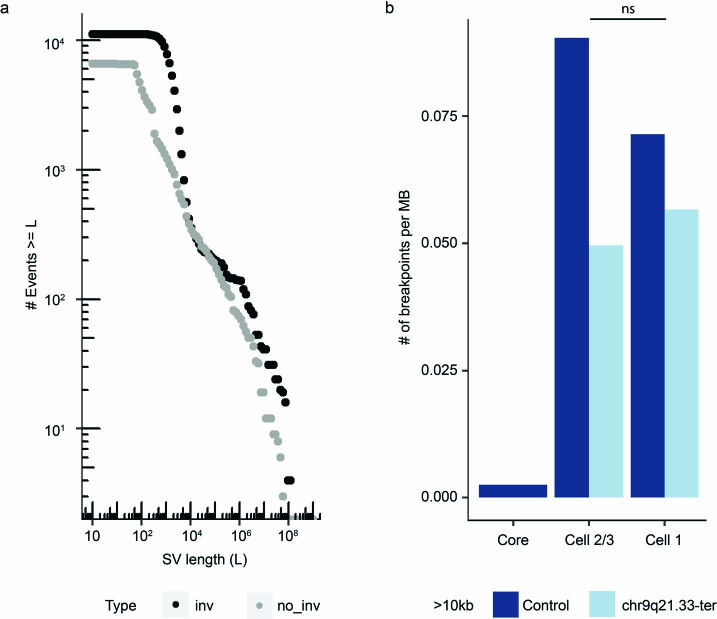
Replication and collective missegregation of Chr.9q21.33-ter did not result in chromothripsis. **a**, Length distribution of inverted and non-inverted structural variants (SVs) in four selected single cells of the dataset presented in Fig. 3 (cell 1 (5 copies of a chr9.q fragment)), cell 2 and 3 (1 copy) and a cell with the core karyotype (diploid)). The enrichment of short-range inverted SVs can be explained by the WGA reaction. A similar count in inverted and non-inverted SVs was observed for SVs with a length ≥10 kb. **b**, The frequency of SVs (length ≥ 10 kb) on all control chromosomes and on the missegregated chr.9q21.33-ter fragment. No enrichment of SVs was observed in the chr.9q21.33-ter region for Cell No. 1 vs. Cell No. 2/3 which suggests that chromothripsis did not occur (p = 1, two-sided Fisher’s exact test).

**Extended Data Fig. 6:**
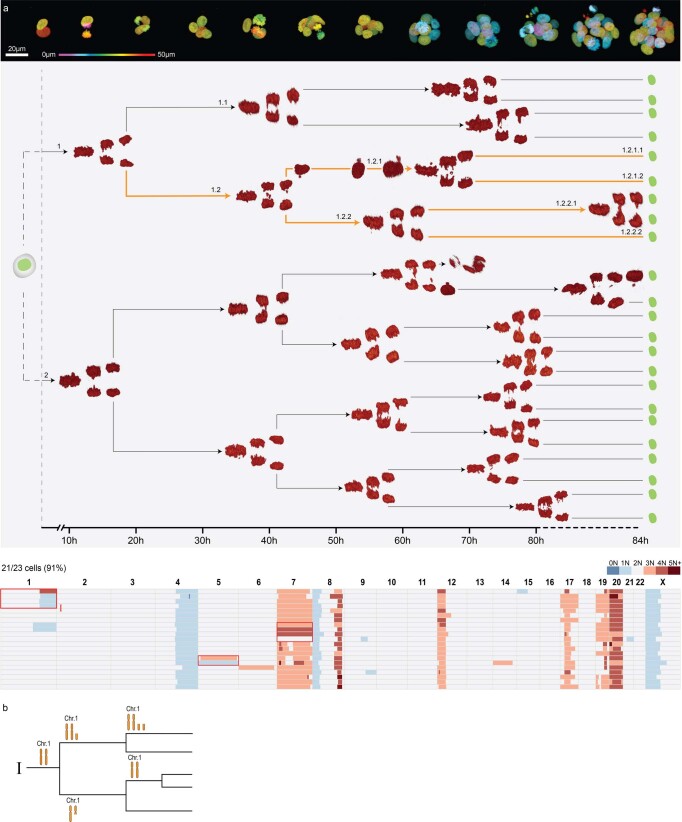
3D Live-Seq dataset of a PDTO-9 organoid consisting of 23 cells. **a**, 3D Live-Seq dataset of a PDTO-9 organoid consisting of 23 cells of which 21 cells were whole-genome sequenced (91% recovery). Top: representative stills of the growing PDTO-9 structure with nuclei in false color depth code. Below: full mitotic tree with 3D-rendered stills of each anaphase. Onset of anaphase is indicated by arrowheads in relation to the time axis (in hours). Bottom panel shows a karyotype heatmap of all cells of the imaged PDTO. Lineage I: one cell carries four copies of Chr1q24.2-ter and three cells carry the reciprocal loss. **b**, Minimal phylogenic structure of lineage I, which only fits branch 1.2 (highlighted in orange) of the mitotic tree. Obtaining four copies of Chr1q24.2-ter involves one round of replication and collective missegregation of the Chr1q fragments (Fig. 3).

We encountered a similar amplification of chromosome 9q (chromosome 9q21.33-ter) within the photoconverted lineage of another PDTO-9 3D Live-Seq dataset (25 cells; 100% recovery), which allowed accurate mapping of its phylogenetic origin across four cell generations (Fig. 3a, lineage I). The amplification of chromosome 9q in one of the photoconverted cells and the reciprocal loss across all other cells within the lineage could only be reconciled by two cycles of replication and collective missegregation of an acentric chromosome 9q fragment (Supplementary Note 3). Chromatin phenotypes along the photoconverted branch suggested that replicated chromosome 9q fragments were missegregated during consecutive divisions 1.1 and 1.1.1, but were probably shielded from a third round of replication before division 1.1.1.1 by micronuclear containment (asterisk), which are known to be associated with replication defects^26–28^. Next, we performed additional deep sequencing of cell 1 (five copies), cells 2 and 3 (one copy) and a cell with the core karyotype (diploid), to extract single-nucleotide variants (SNVs) unique for the chromosome 9q21.33-ter region of each parental chromatid. As expected, the cell containing an amplification of chromosome 9q displayed a biased variant allele frequency of SNVs in favor of one parental allele and at a degree consistent with two rounds of replication (Fig. 3b). Notably, no indications of chromothripsis^21,29^ were found within the amplified chromosome 9q fragment (Extended Data Fig. 5), suggesting that micronuclear envelope integrity was maintained^30^. Similar sub-chromosomal amplifications were observed in additional datasets (Extended Data Fig. 1 (lineages III and IV) and Extended Data Fig. 6, (lineage I). In each case, the amplified sub-chromosomal fragments were acentric, suggesting a model where a lack of spindle microtubule attachment results in non-disjunction of the replicated acentric fragments. During anaphase, the unattached replicated fragments are collectively displaced to one daughter cell, probably resulting in a stochastic nuclear or micronuclear containment. While the impact of amplified acentric fragments on tumorigenesis and tumor evolution is speculative, it is conceivable that amplified acentric chromosomal fragments act as substrates for chromothriptic events, contribute to the formation of extrachromosomal DNA or eventually fuse to chromosomes, resulting in a stable inheritance of the amplified segment.

### Gross karyotype alterations result from multipolar spindle defects

While CNA events generally involve one or a few chromosomes, we identified two cells that displayed extensive reciprocal CNAs across their genomes, and their karyotypes can be classified as hopeful monsters (Fig. 3a, lineage II). In contrast with the population of hopeful monsters that we previously detected in PDTO-19b, these cells displayed an unbalanced distribution of the core karyotype of lineage I without signs of previous genome duplication, demonstrating that near-haploid and near-triploid genomes can be generated in a single division without tetraploid intermediates^20^. The uneven distribution of chromosomes matched the substantial difference in chromatin mass seen between daughter cells of division 1.1.2.2. Furthermore, the irregular shape of the metaphase plate indicated a multipolar spindle defect as a cause of the unbalanced chromosome distribution. Multipolar spindle defects are associated with a loss of spindle pole integrity^31^ or supernumerary centrosomes. Indeed, the distribution of a replicated genome among excess spindle poles is likely to instigate a genome-wide misallocation of chromosomes.

**Fig. 4:**
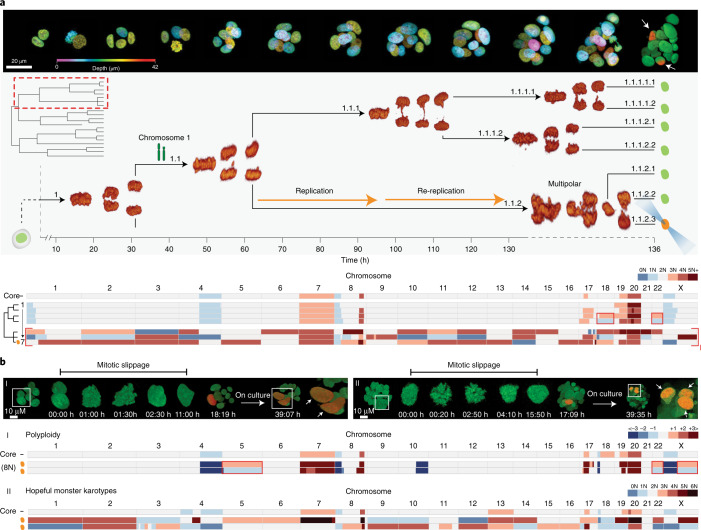
Genome duplication events are substrates for hopeful monster karyotypes. **a**, 3D Live-Seq dataset of a PDTO-9 organoid consisting of 19 cells (95% recovery). Top: representative stills of the growing PDTO-9 structure with nuclei coded in a false color by depth. Photoconverted cells are indicated in the final still (white arrows). One photoconverted cell was mapped to branch 1.1 of the mitotic tree, as highlighted (dashed red box) in the full tree structure at the top left of the middle panel and enlarged in the main image of the middle panel, with 3D-rendered stills of each anaphase. The onset of anaphase is indicated by arrowheads in relation to the time axis. Bottom: karyotype heatmap of all cells mapped to branch 1.1 (all cells share a loss of chromosome 1p; chromosome cartoon) and a reference core karyotype of the imaged PDTO. Three cells, including the photoconverted cell, showed reciprocal hopeful monster karyotypes (I) and were mapped to tripolar division 1.1.2 using the photoconversion reference landmark. Cell 1.1.2 underwent re-replication before anaphase, as indicated by the difference in ploidy between branches 1.1.1 and 1.1.2. **b**, Daughter cells post-mitotic slippage maintain polyploidy of the genome-duplicated ancestor. Lineage I: representative imaging stills (top) of a binucleated cell undergoing mitotic slippage. A PDTO with a photoconverted slippage cell was cultured overnight. The final still shows two photoconverted daughter cells (white arrows) with a similar chromatin mass. Bottom: karyotype heatmap of both daughter cells and the remaining PDTO cells (core). Daughter cells showed polyploidy (8 N) but roughly maintained the core karyotype. Heatmap colour coding indicates deviations with respect to the ploidy of the core karyotype (2N) or daughter cells (8N). The red boxes indicate reciprocal gains and losses. Lineage II: as described for lineage I, but showing a multipolar spindle defect after mitotic slippage resulting in hopeful monster karyotypes among three daughter cells (white arrows). Sequencing data were obtained from two out of three daughter cells and display gross genome-wide karyotype alterations relative to the core karyotype.

Since centrosome amplification is frequently associated with whole-genome doubling^32^, we hypothesized that genome-duplicated cells, resulting from mitotic entry and exit defects, could serve as efficient substrates for the generation of hopeful monster karyotypes by frequently instigating multipolar spindle defects. Indeed, we captured a tripolar division following a re-replication event, which generated three daughter cells with reciprocal hopeful monster karyotypes (Fig. 4a (lineage I) and Supplementary Note 3). Collectively, their genomes aggregate to a twice-replicated core karyotype with a de novo loss of chromosome 1p (1pter–p34.2) (Extended Data Fig. 3b). Cells 1–4 displayed the same loss of chromosome 1p and were mapped to branch 1.1.1. The difference in ploidy between branches 1.1.1 and 1.1.2 and the absence of nuclear envelope breakdown and a prolonged cell cycle time of cell 1.1.2 clearly support re-replication. Mitotic slippage similarly results in a genome-duplicated cell state and, in contrast with re-replication events, can readily be recognized by sequential chromatin condensation and de-condensation without entering anaphase.

**Extended Data Fig. 7:**
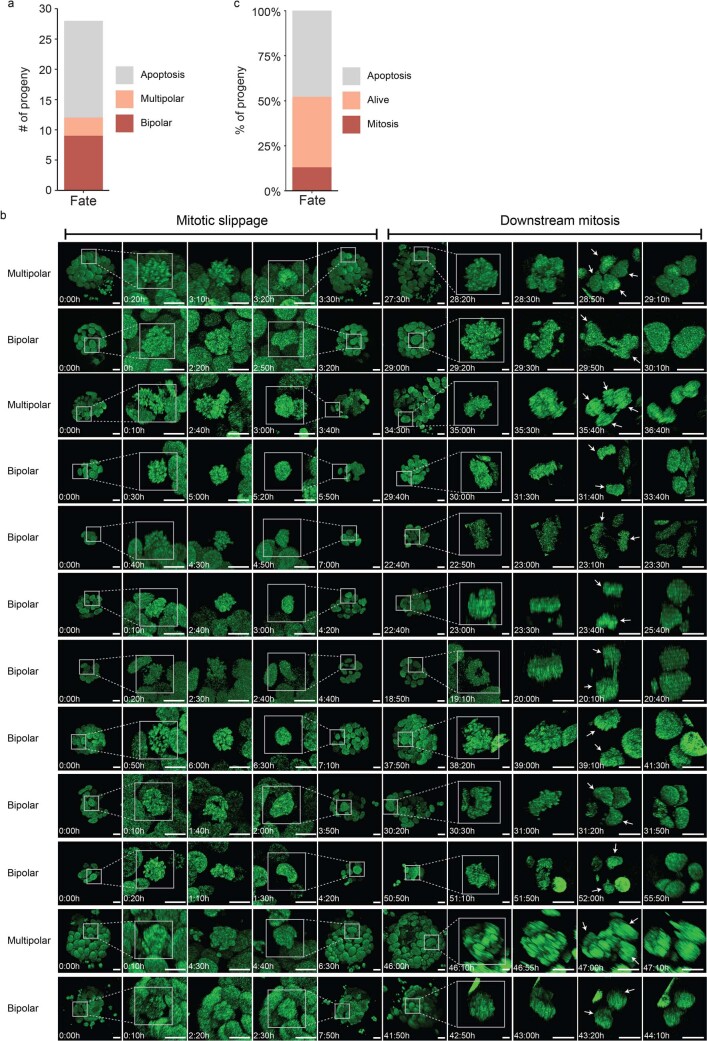
Fate of mitotic slippage events. **a**, Bar graph showing the fate of traced mitotic slippage events in PDTO-9 expressing transgenic H2B-Dendra2. 99 mitotic slippage events were traced, each from independent organoids. 28 slippage events resulted in downstream apoptosis or mitosis and were included in the statistics. Mitotic events were segregated into bipolar or multipolar spindles based on their chromatin phenotype. **b**, Imaging stills tracing the fate of all mitotic cells (n = 12). Spindle polarity is indicated per division. Scalebar is 10μm. White arrows indicate daughter nuclei. **c**, Fate analysis of progeny from 19 multipolar spindle divisions of any origin.

To investigate cell fate after mitotic slippage, we recorded confocal imaging data of PDTO-9 organoids for 72 h to identify and trace mitotic slippage events. Although apoptosis was a more frequent outcome, we scored a high number of cells entering mitosis following a previous mitotic slippage event. Multipolar spindle defects were common among these downstream cell divisions, with 3 out of 12 cells displaying three or more spindle poles (Extended Data Fig. 7a,b). To confirm that multipolar spindle defects resulting from upstream mitotic slippage generate progeny with hopeful monster karyotypes, we photoconverted cells after mitotic slippage and isolated the progeny after their next cell division. As expected, when two daughter cells were generated with equal chromatin mass, we detected polyploidy (including a few reciprocal CNAs) but no genome-wide misallocation of chromosomes (Fig. 4b, lineage I). In contrast, when downstream division resulted in three daughter cells, indicating a multipolar spindle defect, the isolated cells displayed hopeful monster karyotypes (Fig. 4b, lineage II).

Collectively, these data demonstrate that genome duplication events, irrespective of their origin, can act as unstable intermediates in the generation of progeny with gross genome-wide karyotype alterations. In addition, since the majority of cell divisions that follow mitotic slippage appeared fairly normal (bipolar) (Fig. 4b (lineage I) and Extended Data Fig. 7a,b), mitotic slippage may represent an important pathway for whole-genome doubling during carcinogenesis and tumor progression, which is a feature of many human cancers^2,3,33^.

### Cells with novel karyotypes frequently remain proliferative

As presented here, 3D Live-Seq of PDTOs enables the reconstruction of karyotype alterations across multiple consecutive cell generations. It reveals the immediate genomic consequences of CIN (that is, the temporal dynamics and patterns by which new karyotypes are constantly being generated, upon which selection pressures act to shape the genomic evolution of advanced human cancers).

**Extended Data Fig. 8:**
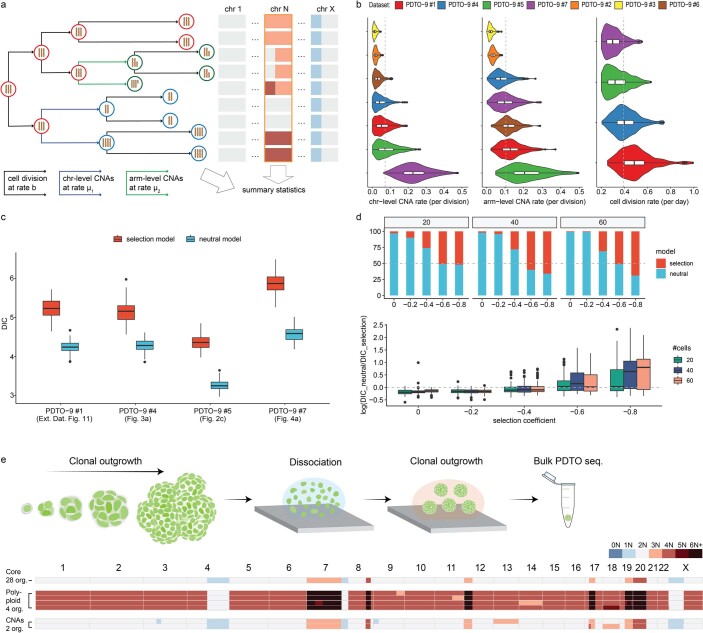
Model selection and cell fate. **a**, Stochastic branching process model of mitosis in a PDTO. Cell division rate = *b* (per day). Per division, de novo reciprocal chromosomal (chr-level) or sub-chromosomal (arm-level) CNAs accumulate at rates *μ*_1_ and *μ*_2_, respectively. Division branches: normal (black), chr-level CNAs (blue), arm-level CNAs (green). Under a selection model de novo CNAs may confer selective (dis)advantages (cells in green and blue). The heatmap on the right shows copy number states of cells in the final population (Grey: 2 N; Light red: 3 N; Dark red: 4 N; Light blue: 1 N). Chr. N copy numbers (orange rectangular box) correspond to chromosomes depicted on the left. Summary statistics were derived from the mitotic tree and copy number states, and used for inference with approximate Bayesian computation (ABC). **b**, CNA rates and cell division rates estimated from seven individual PDTOs (four PDTOs with mitotic trees) using ABC under the neutral model. Violin plots (posterior distributions) and box plots were generated from n = 500 independent samples. Box plots: boxes represent quartiles 2 and 3, horizontal lines represents median; whiskers extend to 1.5× of the interquartile range; data beyond interquartile range are plotted individually. **c**, Model selection using deviance information criteria (DIC, smaller values indicate better support) suggests the neutral model is better supported (100 replicates). Box plot criteria as in **b**. **d**, Power analysis with DIC on the detectability of negative selection in simulated data (n = 100 per parameter setting). Top: number of times each model is supported with different selection strengths and cell numbers. Bottom: differences of DIC values computed under the two models. With increasing strength of negative selection or more cells, the power to detect negative selection increases. Box plots as in **c**. **e**, As in Fig. 5e but for an independent parental organoid. 28 organoids share the reference PDTO-9 core karyotype, 4 were polyploid and 2 had de novo localized CNAs.

Our 3D Live-Seq datasets suggest that intrinsic negative selection against de novo karyotypes, including grossly altered hopeful monster karyotypes, is often not absolute or instantaneous. To derive quantitative evidence for the strength of selection experienced by novel karyotypes, we used a stochastic branching process model to simulate CNA evolution and clonal selection within organoids using cell cycle parameters and measures of karyotype diversity extracted from our datasets. The results were best explained by a neutral drift model, although the power to detect subtle selection pressures was restricted due to the limited dataset size (Extended Data Fig. 8a–d). To further explore selective forces in 3D Live-Seq data, we developed a likelihood ratio test to assess whether there was evidence for a change in birth rate following mitotic events. Using this approach, we were able to aggregate all four mitotic trees of PDTO-9 and found evidence for a decrease in birth rate after a mitotic error (*P* = 0.029; Supplementary Note 4). Taken together, this implied that many de novo karyotypes remain proliferative over a timescale of a few cell generations and experience neutral or subtle negative fitness effects.

### Gross deviations of the core karyotype are generally less fit

**Fig. 5:**
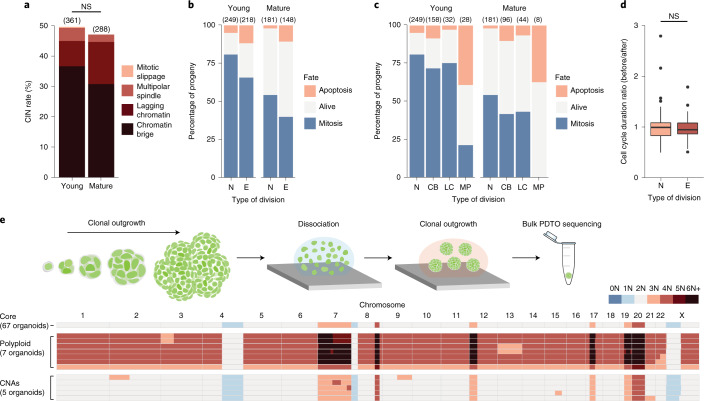
Novel karyotypes frequently remain proliferative and can seed new PDTOs. **a**, CIN phenotype of PDTO-9 expressing transgenic H2B-Dendra2 during early and mature stages of outgrowth. The numbers of scored divisions are indicated in brackets. The total CIN rates were analyzed by chi-squared test (*P* = 0.56; not significant (NS)). **b**, Fate analysis of PDTO-9 progeny resulting from normal (N) or erroneous (E) cell divisions per growth stage. Alive progeny were non-proliferative until the end of the imaging window and this inactive period exceeded the average cell cycle length. The numbers of scored events are indicated in brackets. The difference in apoptotic rates was statistically significant, as determined by chi-squared test (*P* = 0.014 (young) and *P* = 0.0026 (mature)). **c**, As in **b**, but the fate analysis is shown for normal (N), chromatin bridge (CB), lagging chromatin (LC) and multipolar cell divisions (MP). **d**, Ratio between the cell cycle duration before a division and the progeny’s cell cycle duration (averaged) after that division. Data from young and mature PDTOs were pooled and subdivided by mitotic fidelity. The difference in cell cycle duration ratios between normal (94 branches) and erroneous divisions (76 branches) was not significant (data not normally distributed; *P* = 6.104 × 10^−13^ (Saphiro); *P* = 0.64 (two-sided Wilcoxon test)). In the box and whisker plots, the boxes represent quartiles 2 and 3, the horizontal lines represent median values and the whiskers represent minimum and maximum values within 1.5× the interquartile range. The data points indicate outlier values that deviate by more than 1.5× the interquartile range. **e**, Top: schematic of karyotype analysis of successfully formed organoids (bulk) grown from single (fit) cells derived from a parental (clonal) PDTO. Bottom: karyotype heatmaps of sequenced PDTOs (bulk; similar sized) as a proxy for the karyotype of the seeding cell. In total, 67 organoids shared the reference PDTO-9 core karyotype, seven were polyploid and five had de novo localized CNAs.

To investigate cell intrinsic selection pressures against de novo karyotypes in closer detail, we captured a high number of individual cell lineages in PDTOs during early stages of outgrowth (days 4–6; up to ~30 cells in size) and mature stages (days 9–11; hundreds of cells). Overall, the level of CIN remained constant from young to mature organoids (Fig. 5a). Apoptosis was readily detected during both stages of outgrowth and, in agreement with previous reports^7^, we detected elevated levels of apoptosis among the progeny of erroneous cell divisions, in particular after multipolar spindle defects (Fig. 5b,c and Extended Data Fig. 7c). For cells that remained proliferative, the apparent slight shift towards longer cell cycle durations immediately after an error was not significant (Fig. 5d; Wilcoxon test, *P* = 0.64). However, a comprehensive analysis of mitotic tree structures using our derived likelihood ratio test was, again, able to indicate evidence for a decrease in birth rate following mitotic errors (*P* = 5 × 10^−6^; Supplementary Note 4).

Thus, a subset of de novo karyotypes are subject to stringent intrinsic negative selection, with large deviations from the core karyotype originating from multipolar spindle defects being least fit. In contrast, the majority of cells with localized karyotype alterations that result from chromatin bridges or lagging chromatin phenotypes clearly remain proliferative, although these cells seem to experience a subtle fitness cost in the aggregate.

### Cells with novel karyotypes can seed new PDTOs

To functionally probe the fitness of novel karyotypes, we assessed the organoid-forming capacity of single tumor cells derived from one parental clonal PDTO. Subsequent karyotype analysis of the newly formed individual organoids (bulk) acts as a proxy for the karyotype of the seeding single cell and can confirm sustained fitness of de novo CNAs. Indeed, cells with (sub-)chromosomal karyotype alterations and even whole-genome duplications were able to develop into new PDTOs (Fig. 5e and Extended Data Fig. 8e), in line with our general observations, demonstrating that from a cell intrinsic and short-term perspective, some cells with novel karyotypes maintain a high level of fitness.

### Punctuated and localized karyotype alterations are ongoing in mature tumors

**Fig. 6:**
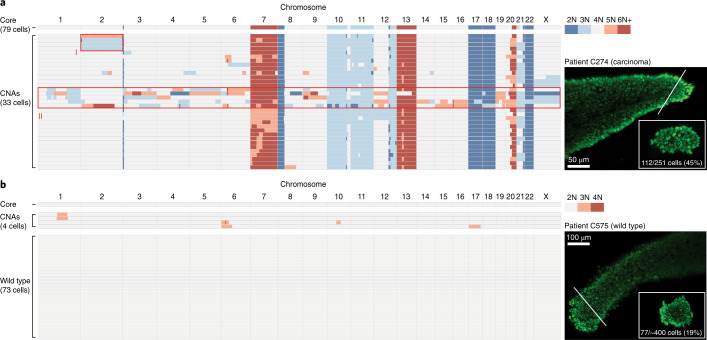
Punctuated and localized karyotype alterations are ongoing in mature CRC. **a**, Karyotype heatmap showing 112 cells (45% recovery) derived from an excised fragment of a single CRC gland isolated from a primary tumor biopsy of patient C274. The remaining gland structure was isolated as bulk (core). A total of 33 cells showed CNAs that deviated from the core karyotype. Glands were stained with Syto 11 to support imaging (right) and single-cell isolation. Reciprocal CNA of chromosome 2 and propagation of the loss is indicated (lineage I). Several cells show gross genome-wide karyotype alterations (lineage II). **b**, As in **a**. A total of 77 single cells were isolated from a wild-type intestinal crypt of patient C575. Four cells showed de novo CNAs of the healthy diploid karyotype. The insets to the right show the excised fragment, obtained by cutting along the solid white lines shown in the main figure.

**Extended Data Fig. 9:**
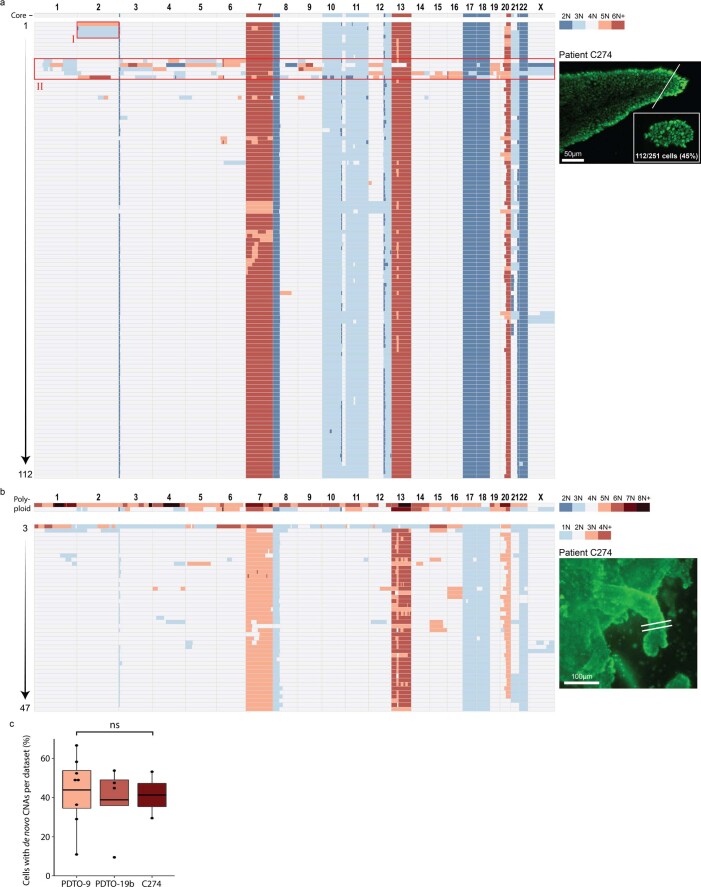
Single-cell sequencing datasets of CRC glands. **a**, Karyotype heatmap showing 112 cells (45% recovery) derived from an excised fragment of a single CRC gland isolated from a primary tumor biopsy of patient C274 (inset). Core: bulk karyotype analysis of the remaining gland structure. (I) Reciprocal loss, gain and propagation of Chr.2. (II) Cells with gross genome wide karyotype alterations. **b**, As in a. 47 single cells were isolated from an excised fragment of a single CRC gland, containing approximately ~500 cells based on fragment size. Two cells in the sequenced population are polyploid. **c**) Box-and-whisker plots of PDTO-9, PDTO-19b and C274 representing the fraction of cells per dataset with de novo CNAs. Boxes represent quartiles 2 and 3, the horizontal line represents the mean and whiskers extend to the minimum and maximum within the 1.5× interquartile range. All data points are shown. Non-significant, p > 0.05 (p = 0.902, one-way ANOVA).

To investigate whether advanced CRCs generate a similar degree of karyotype diversity in vivo before the reshaping of the karyotypic landscape as a consequence of selection, we adapted our sequencing methodology to measure karyotype diversity within clonal lineages of freshly frozen human CRC samples. To maximize recent ancestry within the sampled cell population, we excised small fragments from individual CRC glands, which are analogous to clonal intestinal crypts of the normal epithelium^34^. Subsequent single-cell isolation and prospective genome analyses displayed substantial karyotype diversity between cells, with almost 30% of cells carrying CNAs that deviated from the core gland karyotype—a number that was in agreement with our PDTO data (Fig. 6a and Extended Data Fig. 9). In contrast, only a few single cells from a wild-type intestinal crypt displayed de novo CNAs, supporting the sensitivity of our assay, in agreement with an earlier report on basal CIN levels in healthy colon tissue^35^ (Fig. 6b). Notably, by capturing a large fraction of clonal tumor cells (44%), we detected the emergence and propagation of a de novo CNA in vivo (Fig. 6a, lineage I). Moreover, we readily identified cells with hopeful monster karyotypes (Fig. 6a, lineage II), further demonstrating that the generation of cells with genome-wide karyotype alterations is ongoing at advanced cancer stages.

## Discussion

**Extended Data Fig. 10:**
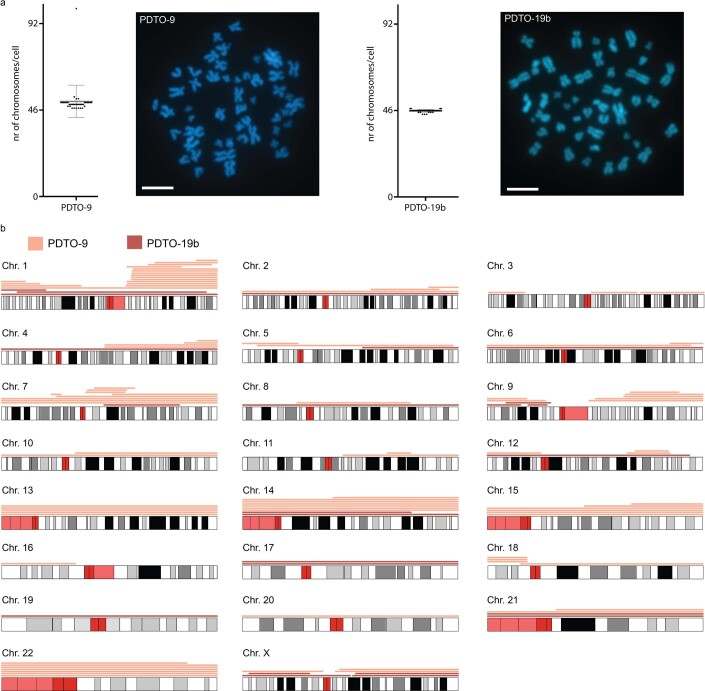
Overview of CNA events per chromosome for PDTO-9 and PDTO-19. **a**, Genome-wide map of all de novo CNA events that were observed in eight PDTO-9 and four PDTO-19b datasets (respectively, 310 and 158 analysed single cells), and 64 PDTO-9 cell divisions (128 analysed single cells). Events include reciprocal CNAs, non-reciprocal whole-chromosome gains or losses and non-reciprocal sub-chromosomal CNAs represented in more than one cell. Hopeful monster karyotypes were excluded for this representation. Centromeric and other complex regions of chromosomes are depicted in red. **b**, Metaphase spread-based karyotyping of 36 tumor cells from PDTO-9 and PDTO-19b stained with DAPI to confirm their near-diploid genome. Each dot represents a tumor cell, center bar and error bars represent the mean and standard deviation. Scalebar is 5 μm.

Our work reveals the genomic consequences of CIN across consecutive cell generations. By reconstructing intermediate genomic states, we demonstrate that both genome-wide (punctuated) and localized (singular, Extended Data Fig. 10) karyotype alterations are ongoing and prevalent in advanced CRC. The substantially reduced fitness of many hopeful monster karyotypes is not surprising given the extent of their karyotype alterations. Nevertheless, low probabilities of increased fitness can still be of potential impact considering that tumors can consist of hundreds of billions of cells^9^. Thus, while most diversity provides incremental variation around a proven-fit aneuploid genome, millions of hopeful monster karyotypes represent extreme, potentially adaptive phenotypes within a fitness landscape. The ongoing generation of karyotype diversity in advanced cancers is in agreement with recent analyses of tumor genomes from large patient cohorts^2,3^. However, as most of these studies measure the net outcome of evolution, it remains difficult to disentangle the rate at which karyotype diversity is generated and the selection pressures that act on them to shape an aneuploid landscape over time. Therefore, our insights into the alteration rate and diversification patterns of tumor karyotypes provide valuable parameters to understand the historic trajectories and temporal dynamics of an evolving aneuploid genome. In addition, our work suggests that aneuploid tumor karyotypes can, in principle, be generated in a single erroneous cell division, providing support for punctuated evolution of malignant karyotypes during the earliest stages of carcinogenesis^13,14,36^.

## Methods

### Patient-derived organoid culture

CRC patient-derived organoids with the identifiers p9T and p19bT were obtained from a previously established biobank and published study^15^. PDTO cultures were maintained at 37 °C with 5% CO_2_ atmosphere, as previously described^15^. The culture medium contained advanced DMEM/F-12 (Gibco) supplemented with penicillin/streptomycin (Lonza; 10 U ml^−1^), GlutaMAX (Gibco; 1×), HEPES buffer (Gibco; 10 mM), Noggin-conditioned medium (10%), R-spondin1-conditioned medium (10%), B-27 (Gibco; 1×), nicotinamide (Sigma–Aldrich; 10 mM), *N*-acetylcysteine (Sigma–Aldrich; 1.25 mM), SB202190 (Gentaur; 10 μM), A83-01 (Tocris; 500 nM) and recombinant human epidermal growth factor (PeproTech; 50 ng ml^−1^). PDTOs were passaged weekly and maintained below passage 10. Briefly, PDTOs were dissociated using trypsin-EDTA (Sigma–Aldrich) and seeded in Cultrex Reduced Growth Factor Basement Membrane Extract (BME), Type 2 in a pre-warmed 24-well plate. ROCK inhibitor Y-27632 (Gentaur; 10 μM) was added to the culture medium upon plating for 2 d.

### Generation of H2B-Dendra2 PDTO lineages

PDTO cultures were transduced with lentivirus carrying H2B-Dendra2-IRES-Puromycin (pLV-H2B-Den2-IRES-Puro was a gift from J. van Rheenen). Cells expressing H2B-Dendra2-IRES-Puromycin were selected by supplementing the culture media with puromycin dihydrochloride (Santa Cruz; 2 μg ml^−1^).

### CRISPR gene tagging

TV-hHIST1H2BC-Dendra2 was generated by Golden Gate assembly^37^ of hH2BC_UHA and hH2BC_DHA (gBlocks; IDT) into TVBB-Dendra2 (Supplementary Table 1). To knock-in Dendra2 at the carboxy terminus of the human *HIST1H2BC* locus, 1 × 10^6^ PDTO-9 cells were co-electroporated with 7.5 μg Cas9 D10A nickase (Addgene; 48141) and 7.5 μg TV-hHIST1H2BC-Dendra2 using the NEPA21 Super Electroporator (Nepagene) following described conditions^38^. A bulk knock-in culture was established by fluorescence-activated cell sorting of a Dendra2-positive cohort 18 d post-electroporation. Site-specific integration was confirmed by a genotyping PCR on bulk genomic DNA extract using locus-specific primer sets (Supplementary Table 1).

### Karyotyping

PDTOs were treated with 0.1 µg ml^−1^ colcemid (Thermo Fisher Scientific) in culture medium for 12 h. Then, PDTOs were dissociated into single cells using TrypLE Express, incubated in a hypotonic 27 mM trisodium citrate solution at 37 °C for 10 min and fixed in methanol:acetic acid solution (3:1). Fixed cells were dropped on a microscope slide, mounted with DAPI-containing Vectashield and imaged on a Zeiss Axio Imager Z1 microscope with a 63× objective. In total, 36 metaphase spreads were imaged for each PDTO line and quantified by manual chromosome counting (Extended Data Fig. 10a).

### Confocal live-cell microscopy and image analysis

To support live-cell microscopy of cell divisions and subsequent photoconversion of daughter cells, PDTO cultures expressing transgenic H2B-Dendra2-IRES-Puromycin were passaged 5–7 d before imaging. PDTOs were harvested 24 h before imaging and resuspended in an ice-cold mix of culture media containing 50% vol/vol BME. The organoid suspension was then seeded in an ice-cold glass-bottom WillCo dish (WillCo Wells) coated with a thin film of BME. PDTOs were allowed to settle on ice before BME polymerization at 37 °C and the addition of culture media. Cells about to go into mitosis were identified by chromosome condensation and imaged until completion of mitosis with a confocal laser-scanning microscope (Leica SP8X, z step 1 µm, 30-s frame rate) equipped with atmospheric and temperature control. Daughter cells of imaged cell divisions were marked via photoconversion of Dendra2 (405 nm laser; 1 min; 10% laser power; per region of interest). Imaging data were analyzed and 3D rendered with Imaris version 9.3 image analysis software (Oxford Instruments).

To support long-term imaging of organoid outgrowth, a dissociated PDTO suspension was filtered twice through a CellTrics 10-μm sieve (Sysmex) to obtain a pure single-cell suspension. Single cells were seeded in BME-coated glass-bottom WillCo dishes as described previously. At 30 h after seeding, the outgrowth of cell doublets was imaged (3-min frame rate; z step = 1 µm). The culture media was refreshed each day to prevent excessive evaporation. Raw data were converted to videos using an ImageJ macro as described^39,40^. Lineage traces were generated manually.

### Spinning disk live-cell imaging

To support long-term imaging of organoid outgrowth using a spinning disk confocal system (Nikon), 3-d-old organoids carrying a *HIST1H2BC*-Dendra2 knock-in or expressing transgenic H2B-Dendra were cultured and seeded in BME-coated glass-bottom WillCo dishes as previously described. The outgrowth was captured for 72 h on days 4–6 and days 9–11 (4.5-min frame rate; z step = 1.4 µm). The imaging data were analyzed with Fiji (ImageJ).

### Single-cell isolation of PDTOs

To generate clonal PDTOs, a dissociated PDTO suspension was filtered twice through a CellTrics 10-μm sieve to obtain a pure single-cell suspension and seeded at medium density in BME. Clonal PDTOs were grown into structures of 60–100 cells before harvesting. Before single-cell isolation, a single PDTO was transferred to a WillCo dish containing phosphate-buffered saline (PBS) (and 1:1,000 Syto 11 (S7573; Thermo Fisher Scientific) for PDTOs lacking H2B-Dendra2-IRES-Puromycin expression) for image acquisition (LSM510; Zeiss). Raw *Z*-stack data were analyzed with Fiji (ImageJ) to obtain the exact cell number, and a 3D render of the imaged organoid was generated using Imaris version 9.3 image analysis software. To isolate single cells, the imaged organoid was mouth-pipetted to a drop of PBS on a siliconized object slide (Sigmacote; Sigma–Aldrich) and mounted on a fluorescence stereo microscope (SMZ18; Nikon) equipped with a Sola LED (Lumencor) for fluorescence-assisted single-cell picking. The organoid was transferred to a 20-μl drop of trypsin-EDTA (Lonza) for dissociation (30 s). After removing trypsin-EDTA, PBS containing 0.5% bovine serum albumin (Sigma–Aldrich) was added immediately. The organoid structure was further dissociated by repeated mouth-pipetting using a 30-μm glass capillary (custom made). Single cells were transferred to PCR tubes containing either 30 μl mineral oil (Affymetrix) or 4 μl H_2_O and each tube was immediately spun down and stored ice-cold. Photoconverted daughter cells were isolated as previously described and visualized with a custom filter set on SMZ18 (FF01-433/24, FF458-Di02 and FF01-482/35). The remaining cells were pooled and transferred to a single PCR tube as a bulk sample. An overview of the single-cell datasets is presented in Supplementary Table 2.

### Organoid-forming capacity of PDTO-9 cells

PDTO-9 organoids were cultured and dissociated as previously described. Single cells obtained from a single parental clonal organoid were reseeded and cultured for 9 d as previously described. Organoids of similar size were isolated and individually bulk sequenced as a proxy for the karyotype of the seeding cell.

### Fresh-frozen CRC tissue specimens

Fresh-frozen tissue samples were collected from University College London Hospitals under ethical approval 11/LO/1613 and via the University College London Hospital Biobank (15/YH/0311) and processed as previously described^41^. All surgically resected samples were collected from patients who had given informed consent.

### Single-cell isolation of CRC glands

Individual tumor glands were mechanically separated from thawed CRC tissue pieces using a fluorescence stereo microscope (SMZ18; Nikon) and transferred to a WillCo dish containing PBS and 1:1,000 Syto 11 (S7573; Thermo Fisher Scientific) for image acquisition (LSM510; Zeiss). A small fragment of individual CRC glands was resected for single-cell isolation. Raw *Z*-stack data were analyzed with Fiji (ImageJ) to obtain the exact cell number of the resected fragment, and a 3D render of the imaged gland was generated using Imaris version 9.3 image analysis software. Single cells of the resected fragment were subsequently isolated as described for PDTOs. The remaining part of the gland was transferred to a single PCR tube as a bulk sample.

### Single-cell sequencing

Single-cell sequencing was performed on clonal PDTOs with and without photoconverted daughter cells and on CRC glands.

#### Single-cell sequencing of clonal PDTOs and photoconverted daughter cells

For cells expressing H2B-Dendra2-IRES-Puromycin, single-cell lysis and whole-genome amplification (WGA) were performed using the REPLI-G single-cell kit (Qiagen) according to the manufacturer’s instructions. A positive control (multiple organoids or HEK293T cells) and negative control (3 µl H_2_O) were included during each WGA reaction. Amplified DNA was purified by phenol:chloroform:isoamyl alcohol (25:24:1) treatment, followed by precipitation with ethanol. To assess the quality of amplified DNA, a multiplex PCR was used to simultaneously amplify nine loci across the human genome in each single cell: *SCYL1*, *PPP5C*, *JMJD6*, *ACTR10*, *ROCK2*, *SCAP*, *SCAMP3*, *SCARB2* and *XPOT*^42^. DNA libraries of each single cell, with two or more amplified products in the multiplex PCR, were constructed with the TruSeq Nano DNA library preparation kit (Illumina). The concentration of all libraries was quantified using a Qubit dsDNA HS Assay kit. Library fragment size profiling was performed using the Agilent TapeStation system using Agilent High Sensitivity D1000 ScreenTapes (Agilent Technologies), and subsequently all libraries with unique indices were pooled equimolarly.

#### Single-cell sequencing of clonal PDTOs and CRC glands

For cells stained with Syto 11, cell lysis was performed with 0.63 mUA protease (Qiagen) and 1× NEBuffer 4 at 50 °C for 2 h, followed by a protease inactivation incubation for 20 min at 80 °C. DNA libraries were prepared from individual cells with a modified KAPA HyperPlus kit protocol (one-seventh of all volumes; Roche). Cleaned-up pooled single-cell libraries were quantified using a Qubit dsDNA HS Assay kit and analyzed using the Agilent 2100 Bioanalyzer HS kit.

Low-coverage whole-genome single-end 75-base pair sequencing was performed on an Illumina NextSeq 500 aimed at generating ~5 million reads per single cell for the WGA TruSeq libraries and more than 100,000 reads for the KAPA libraries. For a subset of WGA TruSeq libraries (cell numbers 1–3 and 15 of the clonal PDTO dataset presented in Fig. 3), whole-genome paired-end sequencing (2 × 150 base pairs) was performed on an Illumina NovaSeq 6000 to a coverage of 15× (cell numbers 2 and 3) or 30× (cell numbers 1 and 15).

### Genomic analysis

Sequence reads were aligned to the human genome reference (hg19/GRCh37) using the Burrows–Wheeler Aligner mapping tool (BWA-MEM; version 0.7.15). Duplicated sequence reads were marked by Sambamba (version 0.6.5) and realigned using the Genome Analysis Toolkit IndelRealigner (GATK; version 3.8), and sequence read quality scores were recalibrated with GATK BaseRecalibrator.

The copy-number status of each single cell was analyzed using Ginkgo^43^. Briefly, the created BAM files were converted to BED format files using BEDtools (version 2.25.0). Then, the aligned reads were binned into 1-Mb variable-length intervals across the genome, normalized and corrected for GC biases, and segmented in regions with an equal copy-number status. Thereafter, a final integer copy-number profile was assigned to each single cell. Single cells that exhibited low-quality sequencing data, defined as high variation between copy-number ratios of segmented regions, were excluded. One exception was made for cell number 9 of dataset 04032020, as this cell clearly belonged to the lineage with segmental loss of chromosome 1p.

To identify high-confidence copy-number variations, regions of at least 25 Mb (excluding bins greater than 5 Mb) with copy-number values deviating >0.6 from the average ploidy were considered to indicate losses or gains. Copy-number variations smaller than 25 Mb were only included if both the loss and gain of that particular region were identified in the clonally expanded organoid, photoconverted daughter cells or CRC gland. To avoid missing copy-number variations, less confident variations (>0.3 and ≤0.6 below or above the average ploidy) with >70% reciprocal overlap between high-confidence variations were reviewed. Heatmaps were generated using the R (version 3.4.3) package ggplot2. All de novo CNA events observed in PDTO datasets are shown in Extended Data Fig. 10b using the R package karyoploteR.

For cell numbers 1–3 and 15 of the clonal PDTO dataset presented in Fig. 3 with 15× or 30× coverage, SNV and indel calling was performed using GATK HaplotypeCaller. The allele frequency of variants located on chromosome 9 (genomic location = 89502430–141213431) in sequences 1 (SC11-20190409_1) and 15 (SC15-20190409_1) (core) were calculated by subtracting all of the variants present on the allele of sequences 2 (SC10-20190409_1) and 3 (SC17-20190409_1) (Fig. 3b). Density plots of the allele frequencies of variants were generated using the R package ggplot2. Manta (version 0.29.5) was used with standard settings to detect structural variants. To exclude structural variants introduced by the WGA (short-range chimeras of the inverted orientation), we established a threshold of structural variant length for which a similar count in inverted and non-inverted structural variants was observed. Next, we estimated the frequency of structural variants (subdivided based on threshold structural variant length) on all control chromosomes and on the missegregated chromosome to determine enrichment of structural variants in the missegregated chromosome.

### Parameter estimation and model selection

We used a stochastic birth–death branching process to model the outgrowth of PDTOs (Supplementary Note 4). Since apoptotic events were rare in the 3D Live-Seq datasets, we modeled only cell birth with rate *b* (per day). During each cell division, *n*_1_ reciprocal chromosome-level CNAs and *n*_2_ reciprocal arm-level CNAs were introduced to daughter cells, where *n*_1_ = ~Pois(*μ*_1_) and *n*_2_ = ~Pois(*μ*_2_). We assumed two models of evolution: a neutral model where all cells have the same fitness; and a selection model in which cells with novel mutation(s) have a selective (dis)advantage, *s*, given by $\frac{{b_{\rm{d}}}}{{b_{\rm{p}}}} = 1 + s$, where *b*_p_ and *b*_d_ are the birth rate of the parent and daughter cells respectively. The model parameters, *θ* = (*μ*_1_,*μ*_2_,*b*,*s*), were inferred using approximate Bayesian computation with sequential Monte Carlo sampling^44,45^. Using simulated data, we validated that the true parameters were inferred accurately. For datasets without recorded mitotic trees, we used single-cell copy-number profiles to estimate mutation rates under the neutral model (assuming *b* = 0.5). For datasets with mitotic trees, we estimated all parameters under both models and selected the model that best fit the data using deviance information criteria^46^. We also examined the power of our approach to detect negative selection at varying selection strengths and cell population sizes. This was done by simulating 100 datasets for each parameter setting, with the parameters estimated from real data (*μ*_1_ = 0.1, *μ*_2_ = 0.1 and *b* = 0.4) using the approximate Bayesian computation rejection algorithm, and computing deviance information criteria under the neutral and selection models for each dataset.

### Statistics and reproducibility

Imaging stills in the manuscript represent (time-compressed) representations of the entire captured imaging data. Representative snapshots of organoid outgrowth were extracted from imaging data at evenly distributed time points across the imaging span. The numbers of samples analyzed are indicated when applicable.

### Reporting Summary

Further information on research design is available in the Nature Research Reporting Summary linked to this article.

## Online content

Any methods, additional references, Nature Research reporting summaries, source data, extended data, supplementary information, acknowledgements, peer review information; details of author contributions and competing interests; and statements of data and code availability are available at 10.1038/s41588-021-00891-2.

## Supplementary information

Supplementary Information Supplementary Notes 1–4

Reporting Summary

Supplementary Tables Supplementary Tables 1 and 2

## Data Availability

BAM files of the single-cell sequencing data are available through controlled access at the European Genome-phenome Archive (EGA), which is hosted by the European Bioinformatics Institute and Centre for Genomic Regulation (https://ega-archive.org) under accession number EGAS00001003812. Data access requests will be evaluated by the University Medical Center Utrecht Department of Genetics Data Access Board (EGAC00001000432) and transferred on completion of an agreement and authorization by the medical ethical committee at the University Medical Center Utrecht at the request of Hubrecht Organoid Technology to ensure compliance with the Dutch Medical Research Involving Human Subjects Act. The copy-number segment calls of all sequenced single cells are publicly available on Zenodo (10.5281/zenodo.4732372).

## Code availability

Full pipeline descriptions and settings used for mapping are available at https://github.com/UMCUGenetics/IAP. Ginkgo, for the analysis and assessment of single-cell CNAs, is publicly available at https://github.com/robertaboukhalil/ginkgo. The code for modeling real data (the stochastic branching process and likelihood ratio test) is freely available at https://github.com/ucl-cssb/CIN_PDO or 10.5281/zenodo.4762533.
