# Supplementary material for: Reconstructing single-cell karyotype alterations in colorectal cancer identifies punctuated and gradual diversification patterns
Source: Nat Genet. 2021 Jul 1;53(8):1187–95. doi: 10.1038/s41588-021-00891-2 (PMC8346364; doi:10.1038/s41588-021-00891-2)
Supplement: Supplementary file 1 — Supplementary Notes 1–4 [file 41588_2021_891_MOESM1_ESM.pdf]

nature portfolio

https://doi.org/10.1038/s41588-021-00891-2

Supplementary information

Reconstructing single-cell karyotype alterations in colorectal cancer identifies punctuated and gradual diversification patterns

In the format provided by the authors and unedited

Supplementary Notes 1

Karyotype heatmaps and imaging stills of cell divisions shown in Fig. 2b

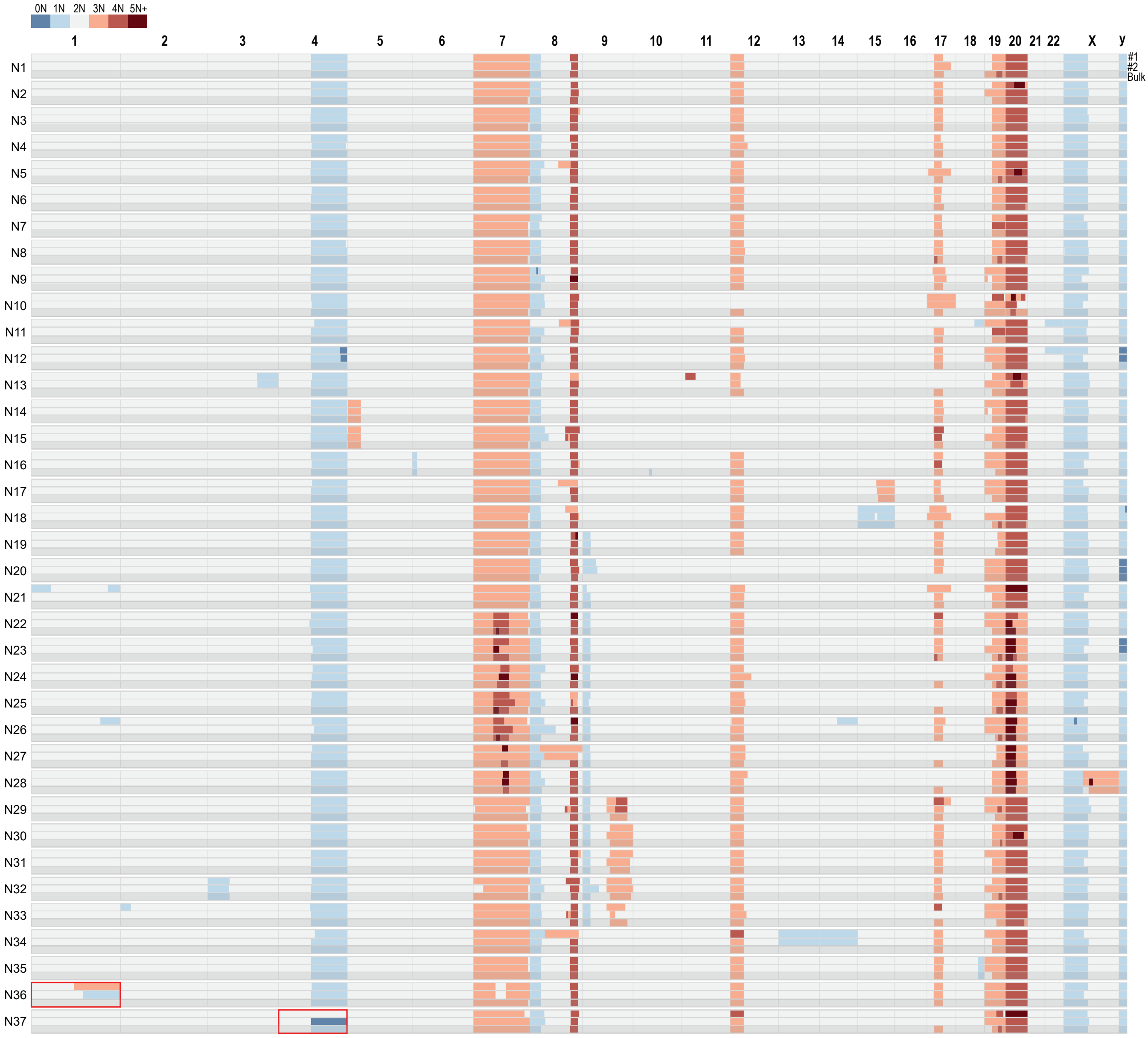

Fig. 1 | Karyotype heatmaps of progeny from cell divisions classified as normal
Karyotype heatmap showing sequencing results of progeny from 37 PDTO-9 cell divisions classified as normal. Daughter cells (#1, #2) are plotted with their respective bulk sample consisting of the remaining cells of the imaged PDTO (darker shade). Reciprocal gains and losses are indicated with red boxes.

Imaging stills of cell divisions classified as normal are shown on page 2-4. Time is indicated in seconds. The scalebar is 10μm

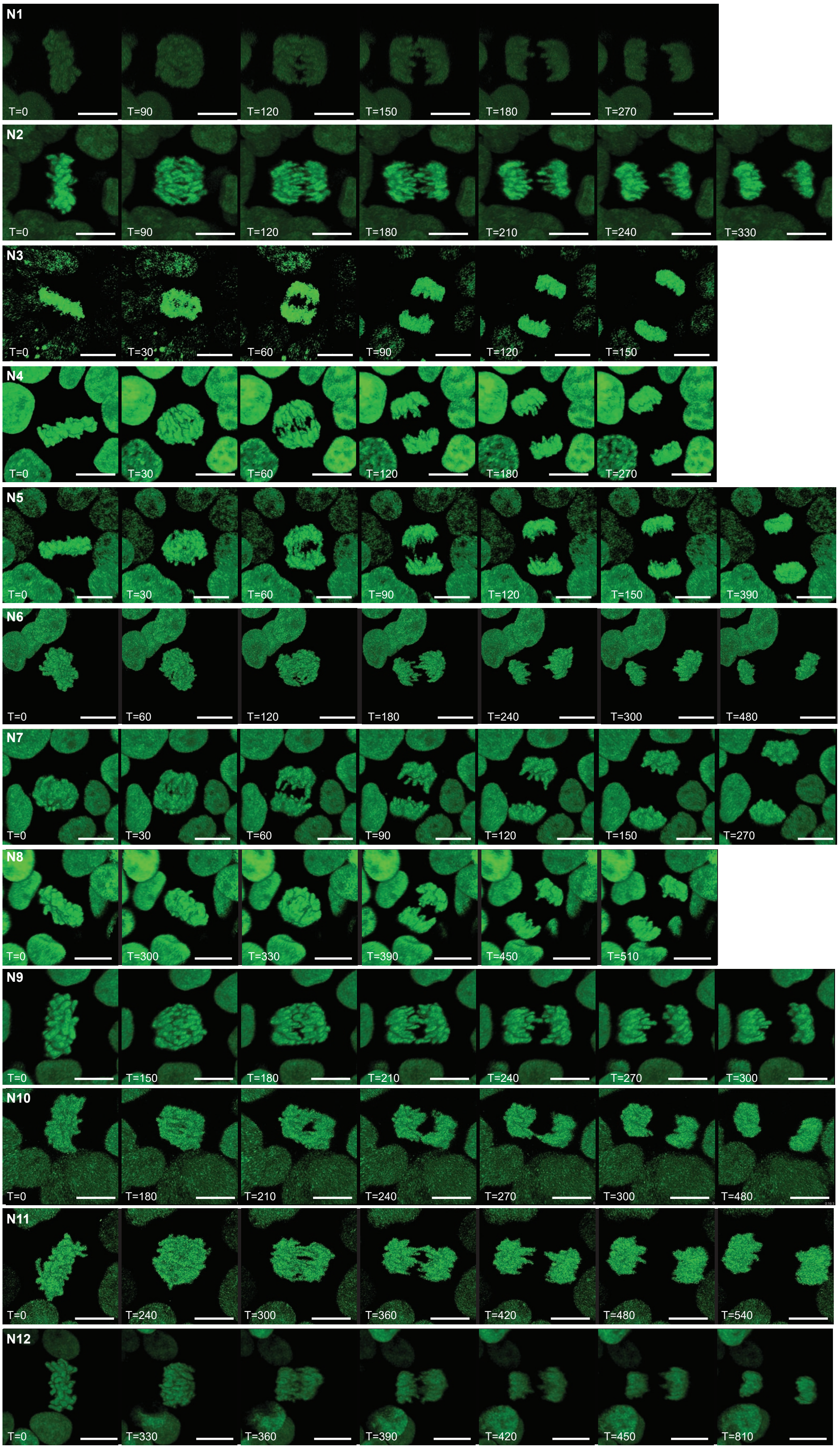
N1
T=0
T=90
T=120
T=150
T=180
T=270
N2
T=0
T=90
T=120
T=180
T=210
T=240
T=330
N3
T=0
T=30
T=60
T=90
T=120
T=150
N4
T=0
T=30
T=60
T=120
T=180
T=270
N5
T=0
T=30
T=60
T=90
T=120
T=150
T=390
N6
T=0
T=60
T=120
T=180
T=240
T=300
T=480
N7
T=0
T=30
T=60
T=90
T=120
T=150
T=270
N8
T=0
T=300
T=330
T=390
T=450
T=510
N9
T=0
T=150
T=180
T=210
T=240
T=270
T=300
N10
T=0
T=180
T=210
T=240
T=270
T=300
T=480
N11
T=0
T=240
T=300
T=360
T=420
T=480
T=540
N12
T=0
T=330
T=360
T=390
T=420
T=450
T=810

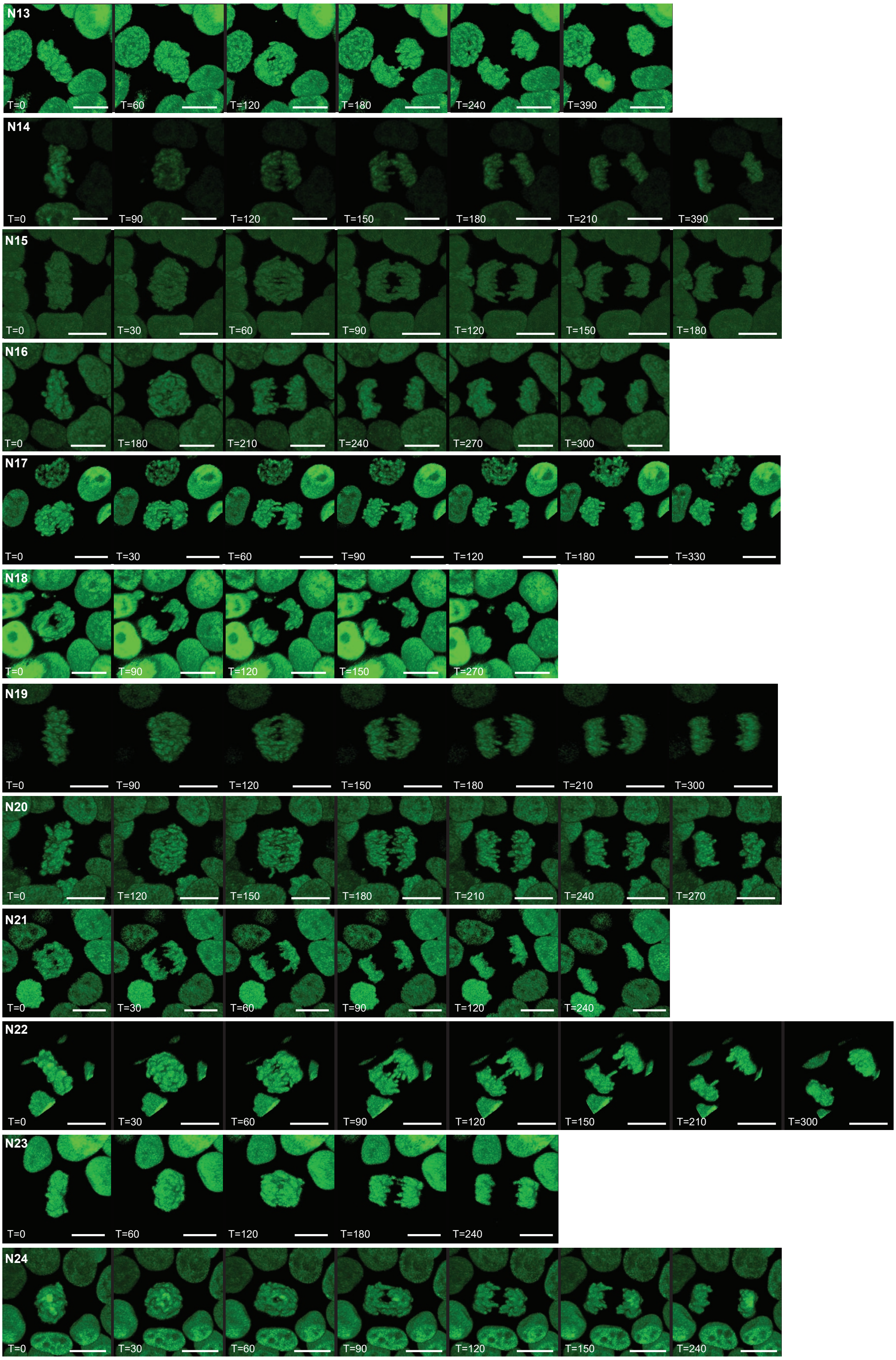

N13
T=0
T=60
T=120
T=180
T=240
T=390
N14
T=0
T=90
T=120
T=150
T=180
T=210
T=390
N15
T=0
T=30
T=60
T=90
T=120
T=150
T=180
N16
T=0
T=180
T=210
T=240
T=270
T=300
N17
T=0
T=30
T=60
T=90
T=120
T=180
T=330
N18
T=0
T=90
T=120
T=150
T=270
N19
T=0
T=90
T=120
T=150
T=180
T=210
T=300
N20
T=0
T=120
T=150
T=180
T=210
T=240
T=270
N21
T=0
T=30
T=60
T=90
T=120
T=240
N22
T=0
T=30
T=60
T=90
T=120
T=150
T=210
T=300
N23
T=0
T=60
T=120
T=180
T=240
N24
T=0
T=30
T=60
T=90
T=120
T=150
T=240

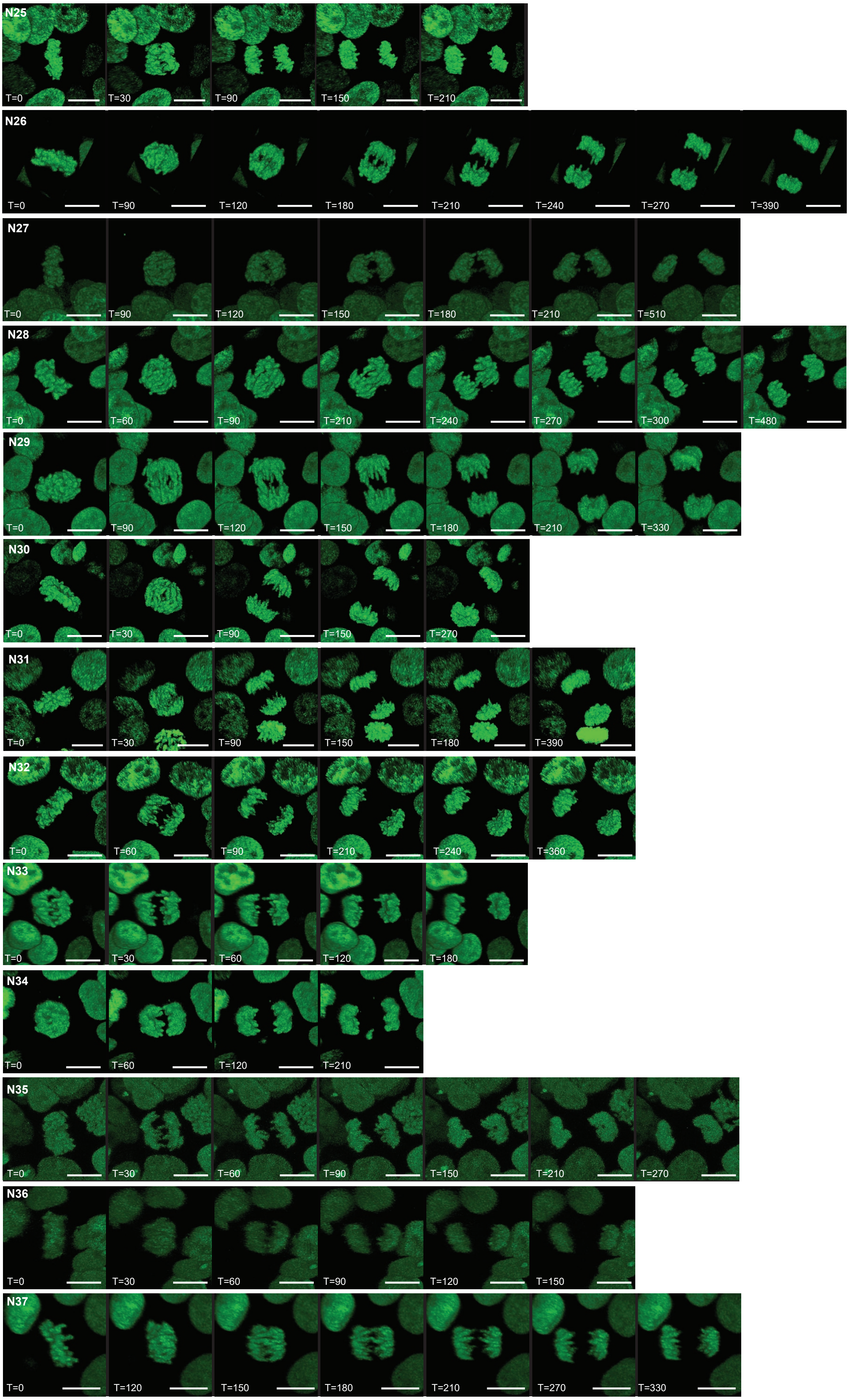

N25
T=0
T=30
T=90
T=150
T=210
N26
T=0
T=90
T=120
T=180
T=210
T=240
T=270
T=390
N27
T=0
T=90
T=120
T=150
T=180
T=210
T=510
N28
T=0
T=60
T=90
T=210
T=240
T=270
T=300
T=480
N29
T=0
T=90
T=120
T=150
T=180
T=210
T=330
N30
T=0
T=30
T=90
T=150
T=270
N31
T=0
T=30
T=90
T=150
T=180
T=390
N32
T=0
T=60
T=90
T=210
T=240
T=360
N33
T=0
T=30
T=60
T=120
T=180
N34
T=0
T=60
T=120
T=210
N35
T=0
T=30
T=60
T=90
T=150
T=210
T=270
N36
T=0
T=30
T=60
T=90
T=120
T=150
N37
T=0
T=120
T=150
T=180
T=210
T=270
T=330

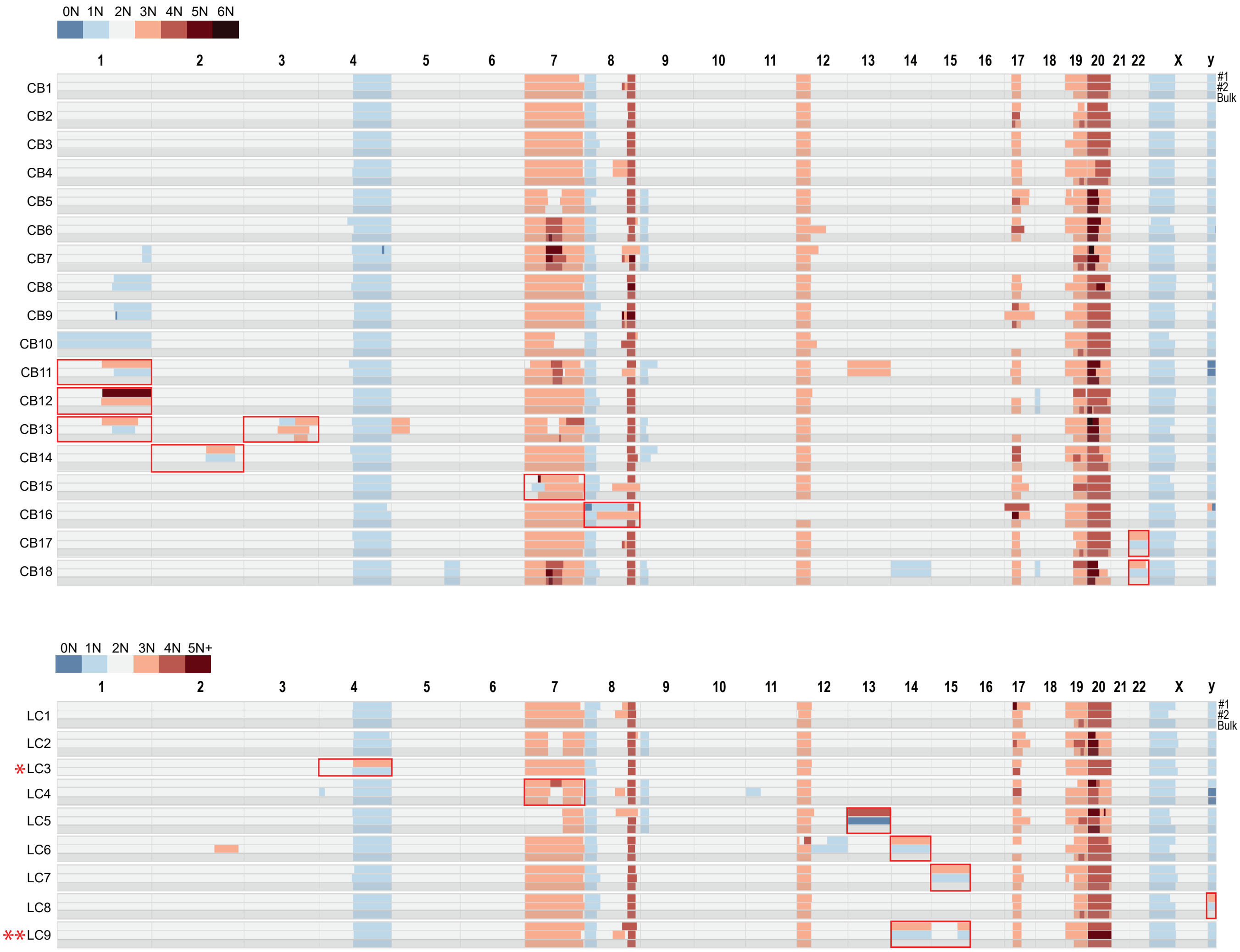

Fig. 2 | Karyotype heatmaps of progeny from cell divisions involving chromatin bridges or lagging chromatin

Karyotype heatmap showing sequencing results of progeny from 18 PDTO-9 cell divisions displaying chromatin bridges and 9 divisions showing a lagging chromatin phenotype. Daughter cells (#1, #2) are plotted with their respective bulk sample consisting of the remaining cells of the imaged PDTO (darker shade). Reciprocal gains and losses are indicated with red boxes. Bulk sample of LC3 is missing (asterisk). LC9 resulted in a sub- and whole-chromosomal missegregation (asterisks).

Imaging stills of cell divisions involving either chromatin bridges or lagging chromatin are shown on page 5-8. Time is indicated in seconds. The scalebar is 10µm

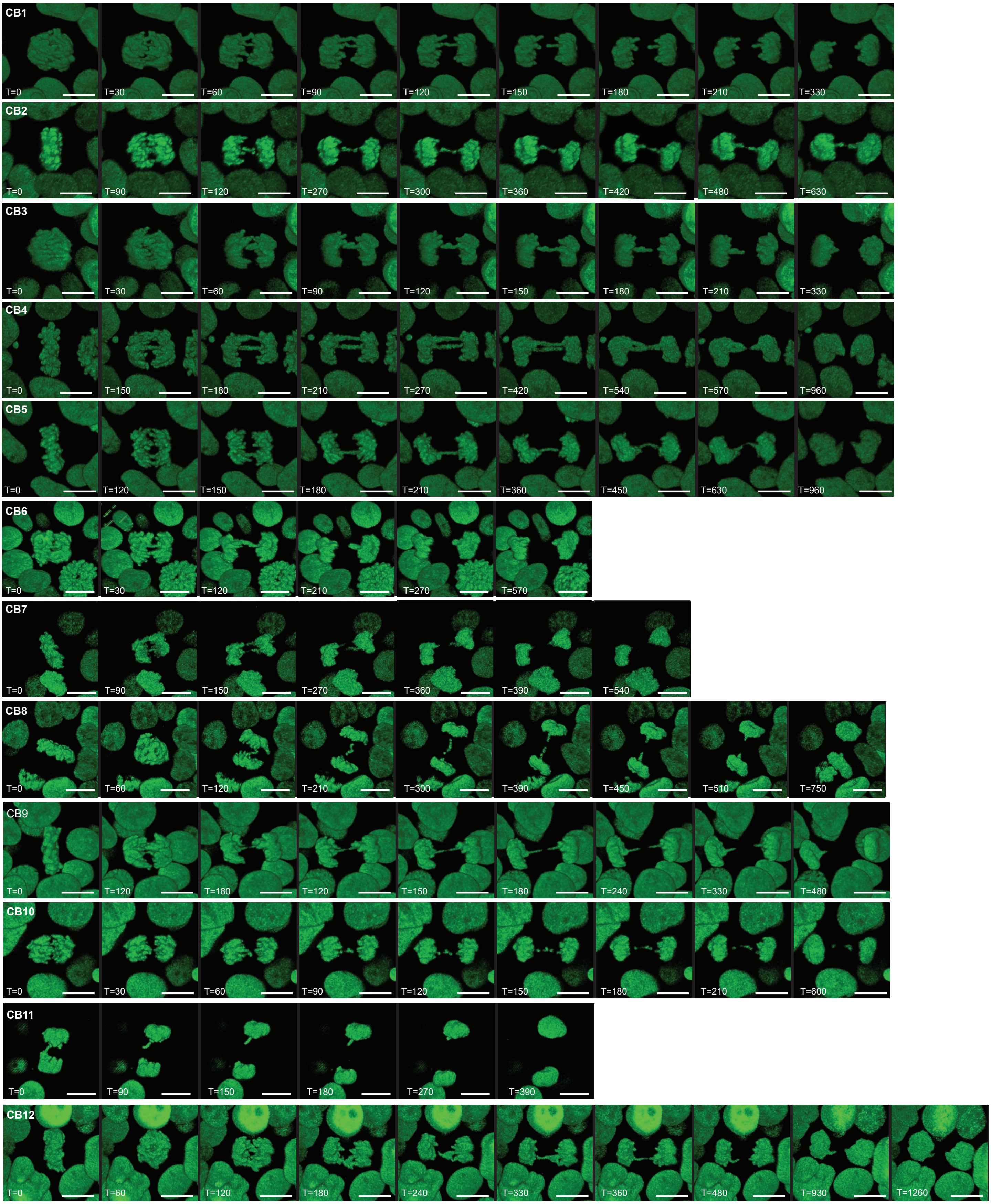
CB1
T=0
T=30
T=60
T=90
T=120
T=150
T=180
T=210
T=330
CB2
T=0
T=90
T=120
T=270
T=300
T=360
T=420
T=480
T=630
CB3
T=0
T=30
T=60
T=90
T=120
T=150
T=180
T=210
T=330
CB4
T=0
T=150
T=180
T=210
T=270
T=420
T=540
T=570
T=960
CB5
T=0
T=120
T=150
T=180
T=210
T=360
T=450
T=630
T=960
CB6
T=0
T=30
T=120
T=210
T=270
T=570
CB7
T=0
T=90
T=150
T=270
T=360
T=390
T=540
CB8
T=0
T=60
T=120
T=210
T=300
T=390
T=450
T=510
T=750
CB9
T=0
T=120
T=180
T=120
T=150
T=180
T=240
T=330
T=480
CB10
T=0
T=30
T=60
T=90
T=120
T=150
T=180
T=210
T=600
CB11
T=0
T=90
T=150
T=180
T=270
T=390
CB12
T=0
T=60
T=120
T=180
T=240
T=330
T=360
T=480
T=930
T=1260

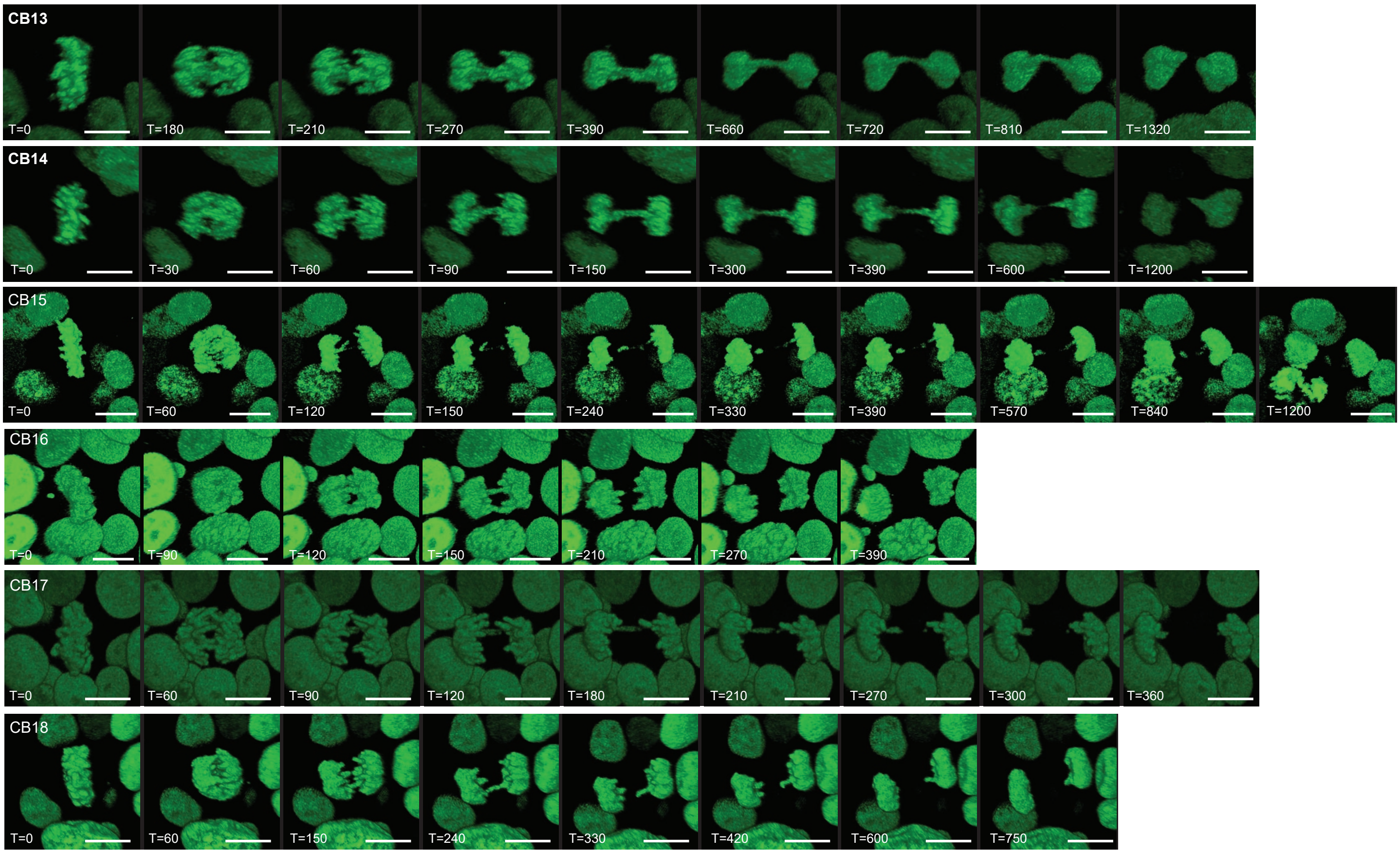
CB13
T=0
T=180
T=210
T=270
T=390
T=660
T=720
T=810
T=1320
CB14
T=0
T=30
T=60
T=90
T=150
T=300
T=390
T=600
T=1200
CB15
T=0
T=60
T=120
T=150
T=240
T=330
T=390
T=570
T=840
T=1200
CB16
T=0
T=90
T=120
T=150
T=210
T=270
T=390
CB17
T=0
T=60
T=90
T=120
T=180
T=210
T=270
T=300
T=360
CB18
T=0
T=60
T=150
T=240
T=330
T=420
T=600
T=750

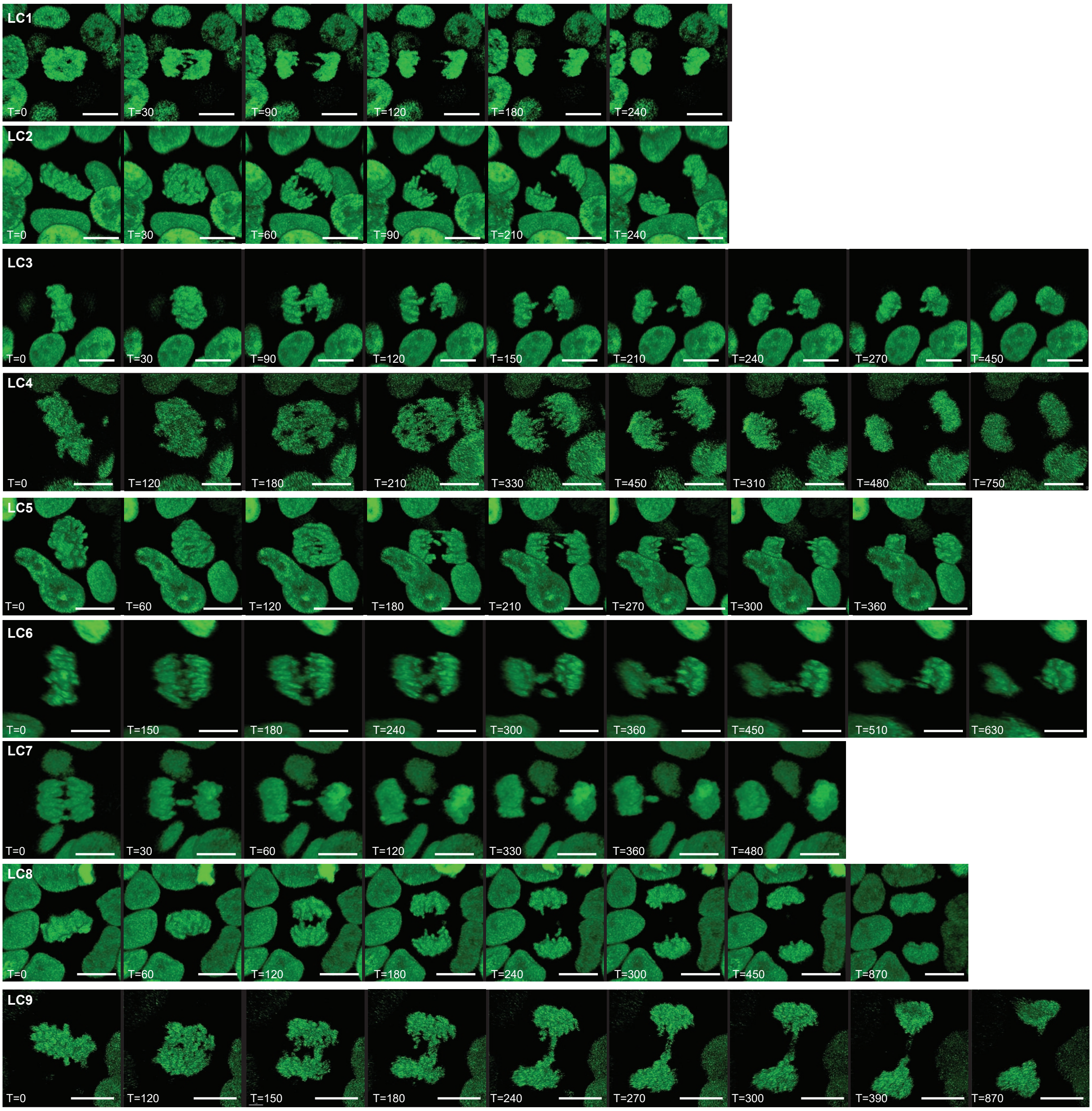
LC1
T=0
T=30
T=90
T=120
T=180
T=240
LC2
T=0
T=30
T=60
T=90
T=210
T=240
LC3
T=0
T=30
T=90
T=120
T=150
T=210
T=240
T=270
T=450
LC4
T=0
T=120
T=180
T=210
T=330
T=450
T=310
T=480
T=750
LC5
T=0
T=60
T=120
T=180
T=210
T=270
T=300
T=360
LC6
T=0
T=150
T=180
T=240
T=300
T=360
T=450
T=510
T=630
LC7
T=0
T=30
T=60
T=120
T=330
T=360
T=480
LC8
T=0
T=60
T=120
T=180
T=240
T=300
T=450
T=870
LC9
T=0
T=120
T=150
T=180
T=240
T=270
T=300
T=390
T=870

Supplementary Notes 2

Detection threshold CNAs

In contrast to artificially induced chromatin bridges in 2D cell lines by Umbreit *et al.*[25], the chromatin bridges that we observe in our tumor organoids often fail to generate detectable CNAs. This discrepancy can be the result of multiple factors.

From a biological point of view:

1. The chromatin bridges as observed by Pellman and colleagues were artificially induced in a 2D cell line (e.g. Nocodazole + RNAi against TP53 and/or CRISPR). Subsequently, large pulling forces could be generated by cells adhered to plastic and generated chromatin bridges which stretched to lengths >200µm which is longer than five times the diameter of average organoids in our manuscript.
2. In contrast to artificial induction of a singular type of DNA error, we observed naturally occurring chromatin bridges in 3D tumor structures that could arise by a plethora of underlying molecular mechanisms. Some are potentially resolved without errors or give rise to CNAs smaller than 10Mb.

From a technical point of view:

3. Umbreit *et al.* have generated higher depth single-cell sequencing data which allows detection of CNAs ≥2.5Mb, whereas we apply a 10Mb resolution throughout our manuscript. However, most CNAs identified by Umbreit et al. were >10Mb, arguing that most CNAs should have been called by our methods as well.

Nevertheless, to compare the sensitivity of both CNA analyses, we have downsampled the single-cell sequencing data of Umbreit *et al*. to match our sequencing depth. Subsequently, we adjusted our detection threshold to ≥2.5Mb like Umbreit *et al*. Reassuringly, our analysis is able to accurately detect previously reported small CNAs (<10Mb) in downscaled data (see figure below). In conclusion, this exercise validates that our analysis, in combination with our data quality, is capable of detecting many of the small CNAs (≥2.5Mb) reported by that Umbreit et al., which may have fallen beyond our conservative detection threshold of 10Mb.

Subsequently, we applied our analysis with the adjusted threshold (≥2.5Mb) to explore whether we could now find previously missed reciprocal CNAs in those daughter cells that originally seemed unaltered (i.e. no CNAs >10Mb). Again, we could not detect smaller reciprocal CNAs that were previously missed, confirming that the absence of CNAs following naturally occurring chromatin bridges is not solely a matter of sequencing depth and CNA size.

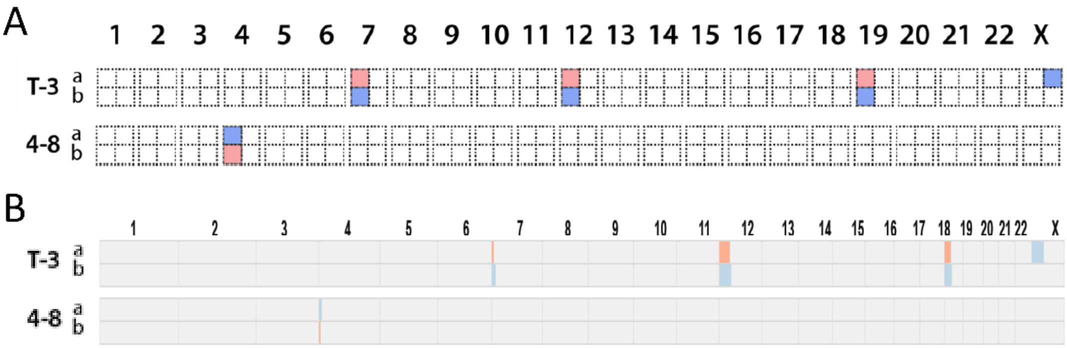

Highly comparable detection of small CNAs by our CNA analysis on downscaled sequencing data and the original report by Umbreit et al.

A) CNA detection according to Umbreit *et al.* for daughter cells originating from an anaphase bridge division (division T3 and 4-8). Dashed boxes show the p- and q-arms of each chromosome.

B) Karyotype heatmap according to our analysis on downscaled sequencing data that equals our sequencing depth, confirms accurate detection sensitivity of reciprocal CNAs between 2.5 and 10Mb in size. Colored copy number state: red, gain; blue, loss. Asterisk denotes CNAs <10Mb.

Supplementary Notes 3

Extended 3D Live-Seq datasets shown in Fig. 3 and Fig. 4a

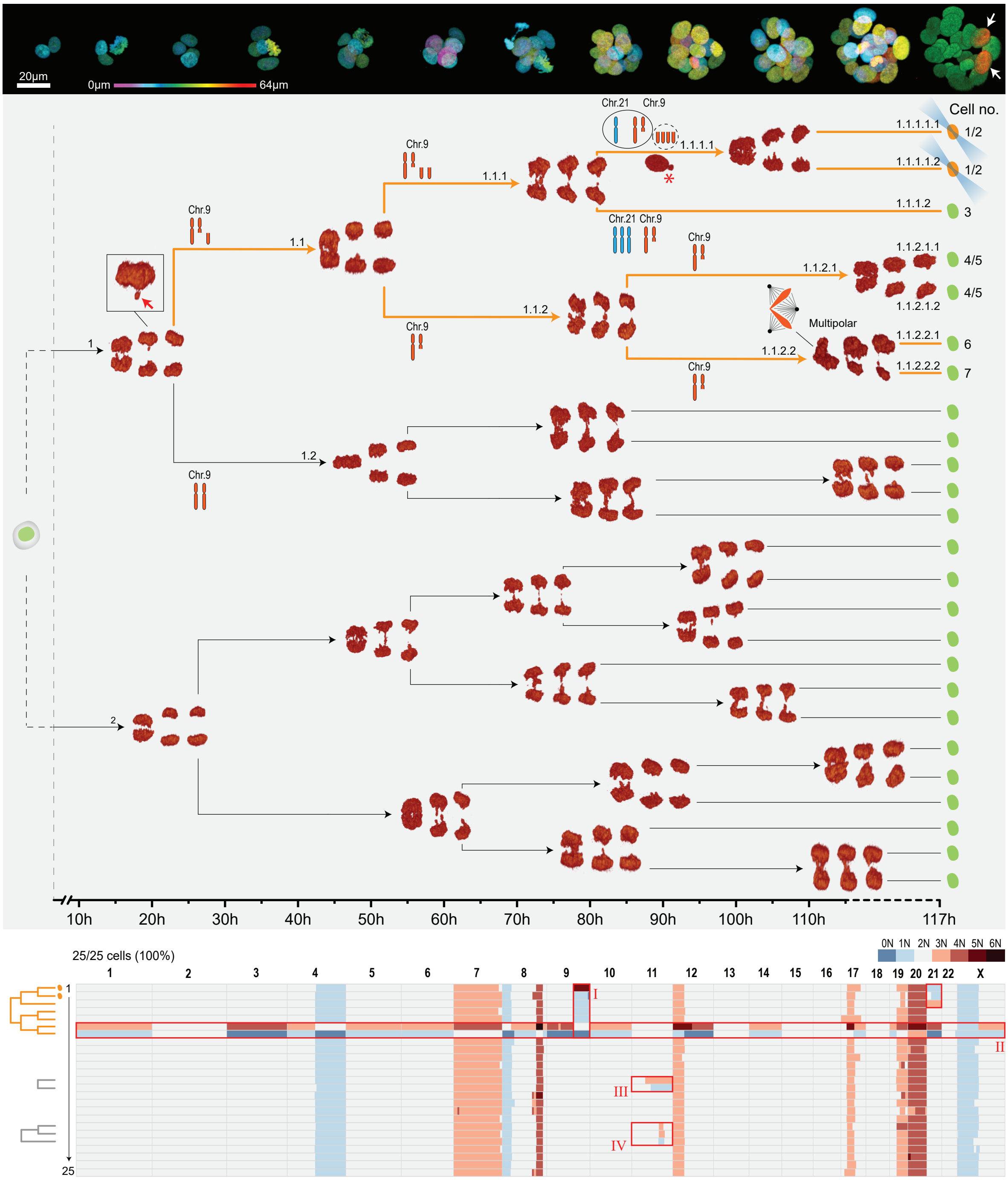

Fig. 1 | Complete 3D live-Seq dataset shown in main Fig. 3
Full 3D Live-Seq dataset of a PDTO-9 organoid shown in Fig. 3 consisting of 25 cells (100% recovery). The top panel shows representative stills of the growing PDTO-9 structure with nuclei in a false color depth code. The final still shows two photoconverted cells (white arrows) that are progeny of branch 1.1 (highlighted) of the mitotic tree. The onset of anaphase is indicated by arrowheads in relation to the time axis (in hours). The bottom panel shows a karyotype heatmap of all cells of the imaged PDTO. Cell numbers of the mapped sequencing results are indicated in the mitotic tree behind a cartoon showing the photoconversion state of each cell. Chromosome cartoons along the mitotic tree indicate copy-number changes across cell generations. The photoconverted cells confine lineage I to the highlighted branch of the mitotic tree. Given the structure of branch 1.1, coherent phylogenetic mapping of lineage I requires two rounds of replication and collective missegregation of a Chr.9q21.33-ter fragment (Extended data Fig. 8). Since both photoconverted cells lost a copy of Chr. 21 (Cell 1 & 2), it follows that cell 3, displaying a gain of Chr. 21, maps to branch 1.1.1.2 and that missegregation of Chr. 21 occurred during division 1.1.1. Chromatin phenotypes along the photoconverted branch suggest that replicated Chr.9q fragments were missegregated during consecutive divisions 1.1 and 1.1.1, but were shielded from a third round of replication prior to division 1.1.1.1 by micronuclear containment (asterisk). The inset of cell division 1 shows a lagging chromatin structure indicated by the red arrow. Hopeful monster karyotypes of cells 6 and 7 (II) are consistent with the difference in chromatin mass between daughter cells of division 1.1.2.2. It follows that the remaining two cells (cell 4 and 5) map to branch 1.1.2.1. The origin of Lineage III and IV cannot be precisely mapped as their phylogenetic solutions are not unique within the mitotic tree.

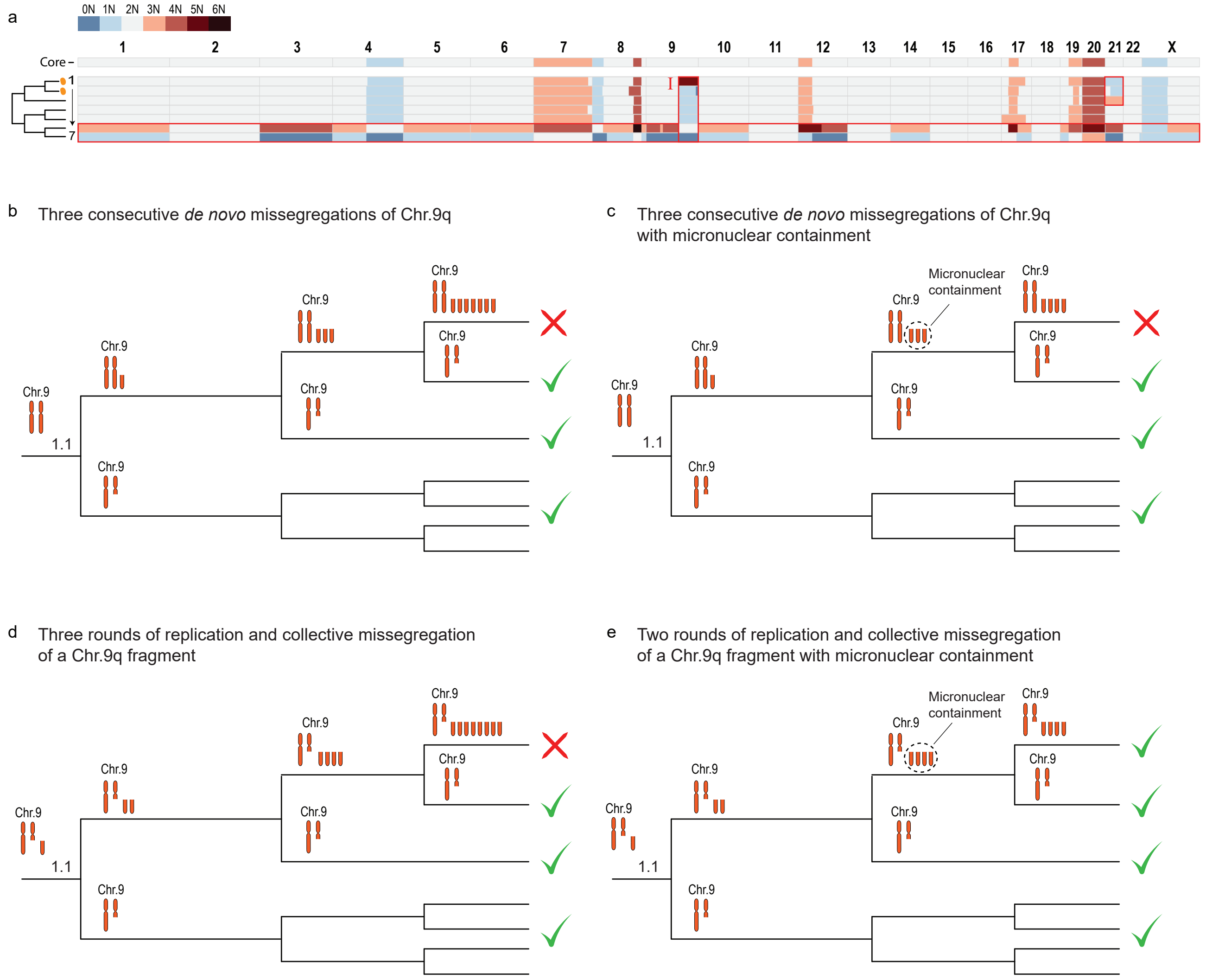

Fig. 2 | Amplification of Chr.9q in main fig. 3 is consistent with two rounds of replication and collective missegregation
a) Karyotype heatmap of lineage I and a representative core karyotype highlighted in Fig. 3. Cell 1 displays 5 copies of the Chr.9q21.33-ter fragment while all other cells in lineage I contain a reciprocal loss of the same region. Note that cell 6 and 7 combined average to a 1 copy state of Chr.9q. Any phylogenetic explanation of lineage I has to comply with the structure of branch 1.1 (Extended data Fig. 7).
b) Three consecutive de novo missegregations of the same parental Chr.9q region is consistent with the copy-number state of cells 2-7 but cannot explain the emergence of a cell with 5 copies of Chr.9q. Note that each de novo missegregation in this model is an identical independent event of the same parental chromatid, which is exceedingly unlikely.
c) As in b, but with micronuclear containment during one cell cycle preventing replication of prior missegregated Chr.9q fragments. This model cannot explain the origin of a cell with 5 copies of Chr.9q.
d) The identical loss of Chr.9q across all branches is consistent with the presence of severed Chr.9q fragments prior to replication and division of cell 1.1. Progeny of cell 1.1 all have an identical Chr.9 core state: carrying one intact Chr.9 chromatid and a Chr.9 chromatid from which a Chr.9q fragment was severed. In this model the replicated Chr.9q fragments are collectively missegregated during three consecutive cell divisions, exponentially amplifying the copy-number. However, three consecutive rounds of replication and missegregation is not consistent with the emergence of a cell carrying 5 copies of Chr.9q.
e) The emergence of a cell carrying 5 copies of Chr.9q is consistent with two rounds of replication and collective missegregation. Micronuclear containment is the most straightforward explanation as to why the Chr.9q fragments were not replicated during one cell cycle.

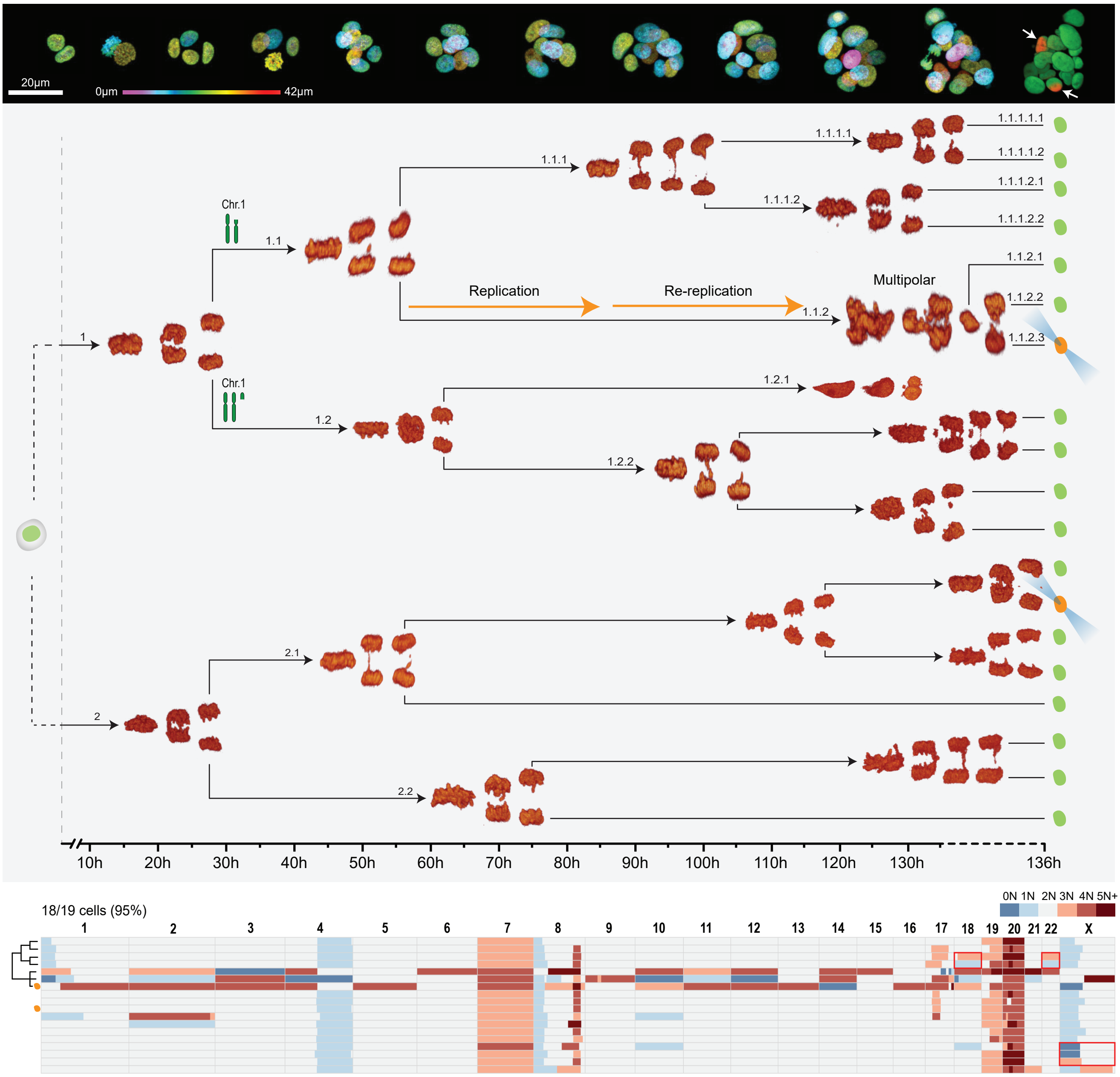

Fig. 3 | Complete 3D Live-Seq dataset shown in Fig. 4a

Full 3D Live-Seq dataset of a PDTO-9 organoid shown in Fig. 4a consisting of 19 cells (95% recovery). The top panel shows representative stills of the growing PDTO-9 structure with nuclei in a false color depth code. The final still shows two photoconverted cells (white arrows) that are part of branch 1.1 and 2.1 of the mitotic tree. The onset of anaphase is indicated by arrowheads in relation to the time axis (in hours). Chromosome cartoons along the mitotic tree indicate copy-number states of historic cell generations. The bottom panel shows a karyotype heatmap of all cells of the imaged PDTO. The three hopeful monster karyotypes (cell 5-7) in this dataset show genome wide reciprocity, and were mapped to tripolar division 1.1.2 by using the photoconversion landmark. Merged hopeful monster karyotypes reveal the presence of a de novo loss of Chr.1p (1pter-p34.2) in the ancestral cell (Extended data Fig. 12). Four other cells share the same de novo loss (cells 1-4) and were mapped to branch 1.1.1. Loss of Chr.1p across branch 1.1 demonstrates initial missegregation of Chr.1p during division 1. Given the propagation of branch 1.2, the absence of cells carrying the reciprocal Chr.1p gain suggests propagation and collective missegregation of the Chr.1p fragment (Fig. 3). The cell receiving the Chr.1p fragments was either not recovered (1 missing cell) or is represented by the apoptotic

Supplementary Notes 4

Branching process modelling of individual organoid tree and sequencing data

We implemented the stochastic birth-death branching process of PDTOs with a rejection-kinetic Monte Carlo algorithm[1,2] in C++. The program is available at https://github.com/ucl-cssb/CIN_PDO.

To infer parameters $\theta = (\mu_1,\ \mu_2,\ b,\ s)$ with approximate Bayesian computation sequential Monte Carlo (ABC SMC)[1,2] we extracted summary statistics $s = (s_1,\ s_2,\ s_3,\ s_4)$ from the data, where s_1 is the average absolute unique chromosome-level copy number changes, s_2 is the average absolute unique arm-level copy number changes, s_3 is half the cell lineage tree (where each node is a cell) length, and s_4 is the average ratio of the waiting time to division of a cell's parent to that of the cell's daughter. To obtain the summary statistics from real data, we performed the following pre-processing. With respect to the mitotic tree, we converted the original time to be in units of a day. We used time on branches between the second-generation anaphase and the last anaphase which are more accurate estimations of cell cycling time. Regarding novel CNAs in each cell, we excluded hopeful monster events which affect multiple chromosomes at a time. We counted all the sub-clonal whole chromosomal events (reciprocal, single or recurrent). For sub-clonal segmental events, we excluded those appearing only once and taking up less than 80% of their corresponding arm. The summary statistics of all the datasets are shown in Supplementary Table 1 (s_4 cannot be computed for dataset PDTO-9 #5 due to the lack of three consecutive reliable branches). We used Euclidean distance to compare summary statistics of simulated and real data. Because s_3 and s_4 have a wide range, we used their natural logarithm values and assigned a weight $w_3\,(=\ 2)$ to $log(s_3)$ for distance computation

on datasets with mitotic trees. The posterior distributions of overall mutation rates (sum of chromosomal-level and arm-level CNA rates) are shown in Supplementary Fig 1. The mutation rates estimated on the three datasets without mitotic trees are consistent with average CNA events per division computed based on data shown in Fig. 1d (Supplementary Fig 2).

We used the function ABCSMC in the Julia package ApproxBayes (https://github.com/marcjwilliams1/ApproxBayes.jl) to run the ABC SMC algorithm. We set prior distributions of parameters to be: $\mu_1 \sim Unif(0,1)$, $\mu_2 \sim Unif(0,1)$, $b \sim Unif(0.2,\ 2)$, and $s \sim Unif(-1,1)$. We took 500 samples to get the posterior distributions. We set target tolerances as below: 0.2 for dataset PDTO-9 #1, #4, and #7, 0.1 for dataset PDTO-9 #5 and 0.015 for dataset PDTO-9 #2, #3 and #6. Other important parameters in the algorithm were set to be default. The algorithm stopped when the target tolerance was reached.

For model selection, we computed deviance information criteria (DIC) based on posterior predictive distributions of parameters[3]. The formula of DIC we used is:

$$DIC = 2\bar{D} - D(\bar{\theta}),$$

where

$$D(\bar{\theta}) = -2\,log(\frac{1}{n}(\sum_{j=1}^{n} K(s - s_j^*)),$$

$$\bar{D} = -\frac{2}{m}\sum_{i=1}^{m} log(\frac{1}{n}(\sum_{j=1}^{n} K(s - s_j^*)),$$

$$K(s - s_j^*) = \frac{1}{\sqrt{2\pi}}\, e^{-\frac{(s-s^*)^2}{2}}.$$

Here, s^* is the simulated summary statistics and $\bar{\theta}$ is the posterior mean of θ. We computed DIC 100 times for each dataset, with $m = 100$ and $n = 100$.

For the power analysis on simulated data, we used the ABC rejection algorithm due to computational efficiency. We set the prior distributions of parameters to be:

$\mu_1 \sim Unif(0,\ 0.5)$, $\mu_2 \sim Unif(0,\ 0.5)$, $b \sim Unif(0.3,\ 0.5)$, and $s \sim Unif(-1, 0)$. To get the posterior distributions, we took the parameter values of the top 1% of 100,000 simulations ordered by Euclidean distance to the true data under each model. We computed DICs one time for each dataset under both models ($m\ =\ 100$, $n\ =\ 100$).

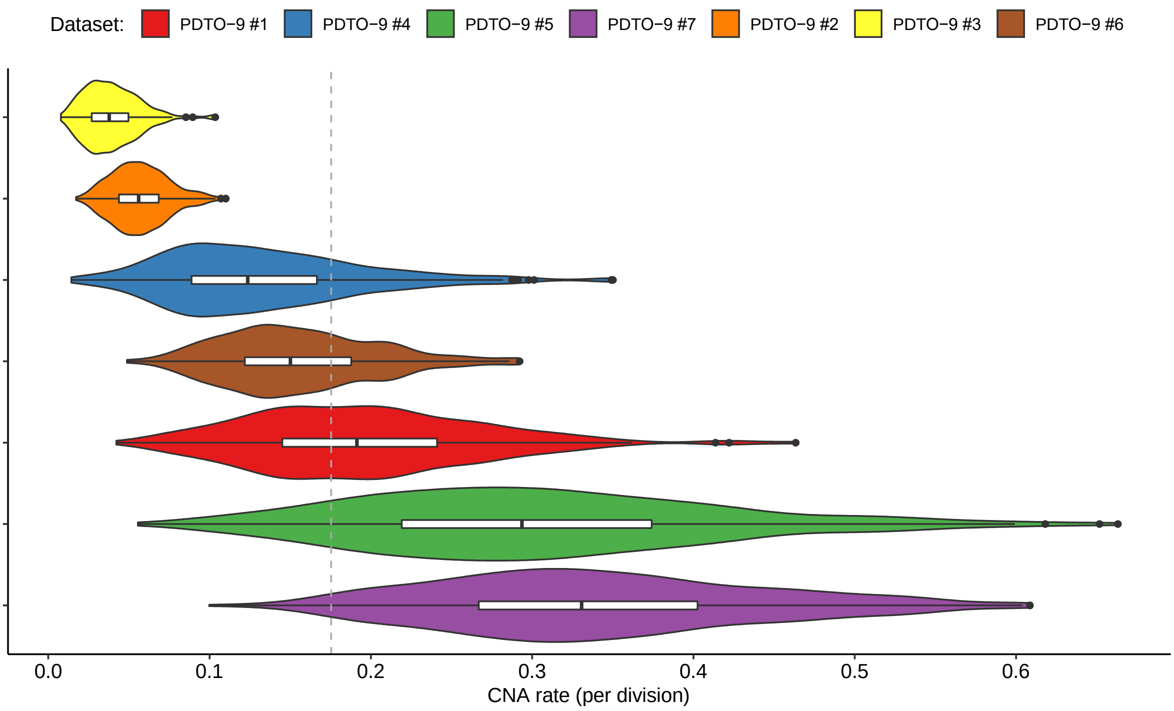

Supplementary Figure 1. Violin plots showing the estimated CNA rates for PDTO-9 datasets. Grey dashed line: weighted mean of estimated rates. The violin plots (posterior distributions) and box plots were generated from n=500 independent samples. The box shows the median (centre), 1st (lower hinge) and 3rd (upper hinge) quartiles of the data; the whiskers extend to 1.5× of the interquartile range (distance between the 1st and 3rd quartiles); data beyond the interquartile range are plotted individually.

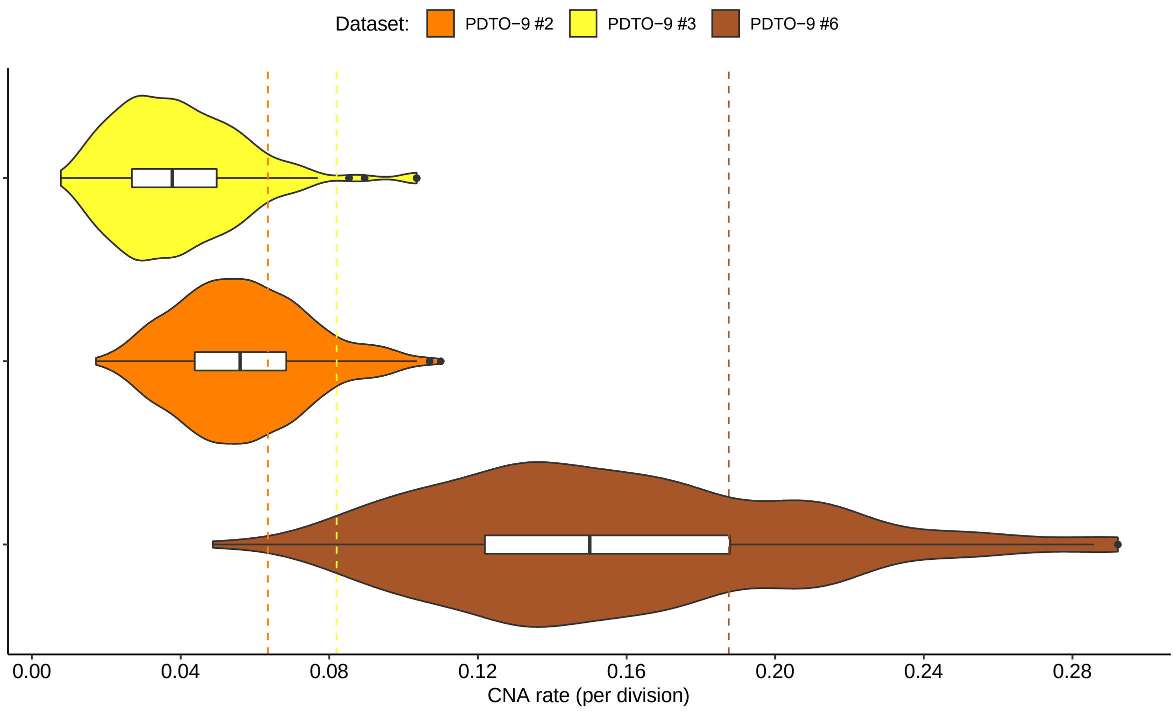

Supplementary Figure 2. The CNA rates estimated using ABC SMC are consistent with estimations based on Fig. 1d. The coloured dash line corresponds to average CNA event per division for each dataset. There are 4 CNA events in n=64 cells (excluding cells not sequenced, 63 divisions) in PDTO-9 #2. There are 5 CNA events and in n=62 cells in PDTO-9 #3. There are 6 CNA events in n=33 cells in PDTO-9 #6. The violin plots (posterior distributions) and box plots were generated from n=500 independent samples. The box shows the median (centre), 1st (lower hinge) and 3rd (upper hinge) quartiles of the data; the whiskers extend to 1.5× of the interquartile range (distance between the 1st and 3rd quartiles); data beyond the interquartile range are plotted individually.

Supplementary Table 1. The summary statistics of PTDO-9 datasets.

**Dataset**	s_1	s_2	s_3	s_4
PDTO-9 #1	0.14	0.43	19.65	1.11
PDTO-9 #4	0.08	0.24	25.99	1.02
PDTO-9 #5	0.17	0.67	14.57	-
PDTO-9 #7	0.71	0.29	25.89	0.89
PDTO-9 #2	0.06	0.11		
PDTO-9 #3	0.03	0.06		
PDTO-9 #6	0.06	0.39		

Yule-tree modelling of the mitotic trees

Due to the low number of cells, there was reduced power to detect a signal of selection in individual mitotic trees. Therefore, we decided to derive a likelihood-ratio test (LRT) on the joint trees to explore if there is evidence of a change of birth (cell division) rate after a mitotic error. The estimations with ABC on dataset PDTO-9 #1, #4, #5 and #7 suggest that the birth rates are similar across datasets. Therefore, we assume the birth rate is constant (b_0) under hypothesis H_0. The alternative hypothesis H_1 is that the birth rate b_0 changes to b_1 after a mis-segregation event which may lead to fitness changes of descendent cells (Supplementary Fig 3). We derived the likelihood of a tree based on the assumption of a pure birth process[4]. Under H_0, the likelihood of tree T with n tips and branch lengths $l = (l_1, l_2, \dots l_{2n-2})$ given birth rate b_0 and present time t is given by

$$L_0(l, n \mid b_0, t) = (n-1)!\, b_0^{n-2} e^{-b_0 \Sigma l},$$

with the maximum likelihood estimate (MLE) of b_0 being

$$MLE(b_0) = \frac{n-2}{\Sigma l}.$$

Under H_1, the likelihood of a tree T with n tips and branch lengths l given birth rate b_0, b_1, and present time t is

$$L_1(l, n \mid b_0, b_1, t) = L_1(l^0, l^1, n, n_0 \mid b_0, b_1, t) = (n-1)!\, b_0^{n_0} b_1^{n-n_0-2} e^{-b_0 \Sigma l^0 - b_1 \Sigma l^1},$$

where n_0 represents the number of internal nodes (birth events, excluding root) before mis-segregation, l^0 represents the lengths of branches before mis-segregation, and l^1 represents the lengths of branches after mis-segregation. Hence, under H_1,

$$MLE(b_0) = \frac{n_0}{\Sigma l^0}$$

and

$$MLE(b_1) = \frac{n - n_0 - 2}{\sum l^1}.$$

The likelihood ratio is

$$LR = -2ln(\max(L_0)/\max(L_1)),$$

where $\max(L_0)$ is the maximised value of $L_0(l, n \mid b_0, t)$ and $\max(L_1)$ is the maximised value of $L_1(l, n \mid b_0, b_1, t)$. Under H_0, the LR asymptotically follows a chi-square distribution with one degree of freedom (df), which is also confirmed by our simulations with R library ape[5] (Supplementary Fig 4).

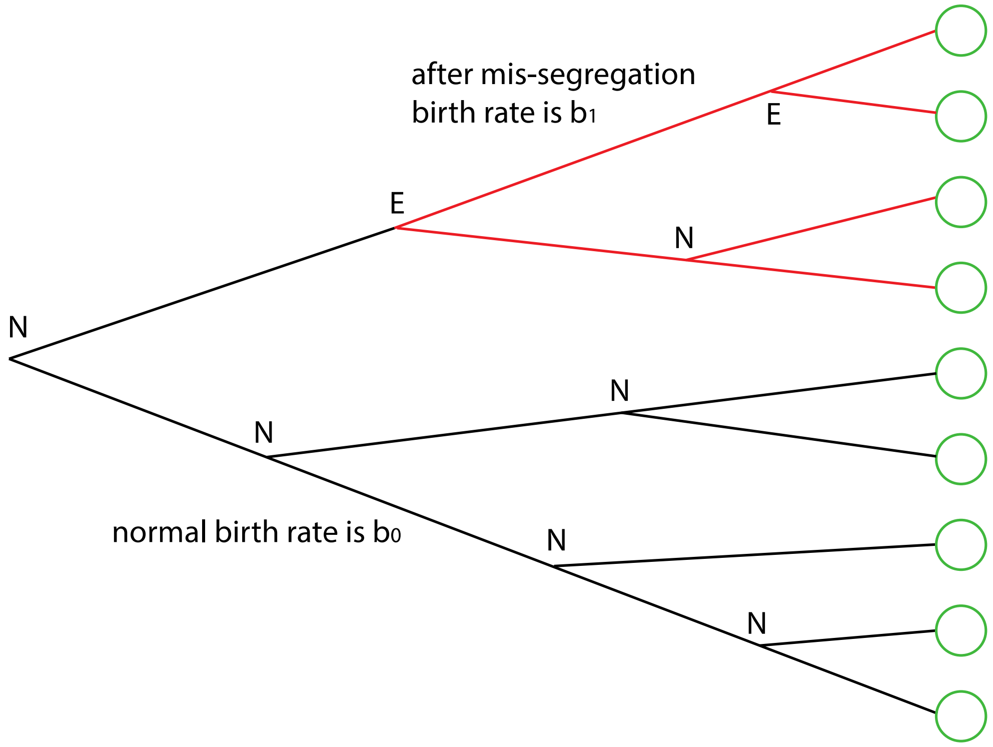

Supplementary Figure 3. The illustration of H_1 in likelihood-ratio test on a pure birth tree. The internal node in the tree represents a cell division event, which is denoted by "N" if it is normal and "E" otherwise. The green nodes represent alive cells at present. We assume the birth rate is b_0 if there is no mis-segregation. Once a mis-segregation occurs, we assume the birth rate becomes b_1, which affects all the decedent cells even if one subsequent division is normal.

Because the PDTO-9 #1, #4, #5, and #7 have an average measurement time of about 5 days and the 60 additional trees has an average measurement time of about 3 days, we applied LRT separately on these two sets of data (Supplementary

Fig 5). For the first batch of four trees, we excluded the eight basal branches whose time measurement tends to be shorter than a complete cell cycle. The results are shown in Supplementary Table 2.

Supplementary Table 2. The results of likelihood-ratio test on real data sets.

**Data set**	$MLE(b_0)$	*max*(L_0)	$MLE(b_0)$ (H_1)	$MLE(b_1)$ (H_1)	*max*(L_1)	*statistics*	*p-value*
4 trees	0.430	42.991	0.627	0.356	45.376	4.770	0.029
60 trees	0.496	-82.360	0.685	0.399	-71.998	20.725	5.3e-6

We performed simulations to quantify the power of our approach in detecting birth rate changes of 0.25 ($b_0 = 0.65$ and $b_1 = 0.4$) on individual trees, 4 joint trees, and 60 joint trees (Supplementary Table 3). We simulated trees under H_1 with time t being 3 and 5 respectively (Supplementary Fig 6). For each parameter setting, we generated 100 datasets (a dataset included m trees when m joint trees were considered) and applied LRT on each dataset. Then we counted the proportion of correct cases when H_0 is rejected (p-value < 0.05) and the estimated b_1 is smaller than b_0. We computed mean power as the mean proportion of correct cases across 10 replicates. As expected, the results suggest the power increases for trees with longer time and a larger number of joint trees.

We also checked the distribution of branch lengths before and after mis-segregation in the simulated data under H_1 (Supplementary Fig 7). We simulated 4 joint trees until time 5 and 60 joint trees until time 3 with $b_0 = 0.65$ and $b_1 = 0.4$. As can be clearly seen with these high statistics, the branch lengths are well described by the model fit.

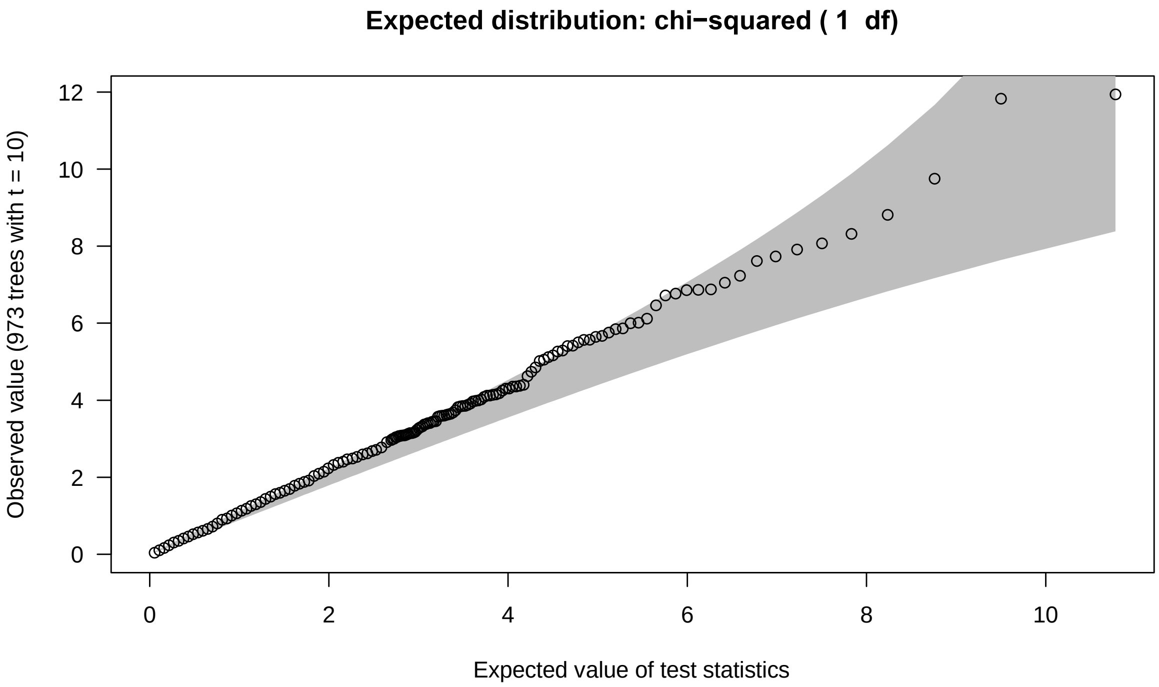

Supplementary Figure 4. The distribution of likelihood-ratio test statistic under H_0. We simulated 1000 random trees with $b_0 = 0.5$ and $t = 10$. For each tree, we assume one subtree is normal and the other is after mis-segregation event. If either subtree has just two tips, we discarded this tree because there is not enough data to estimate birth rate.

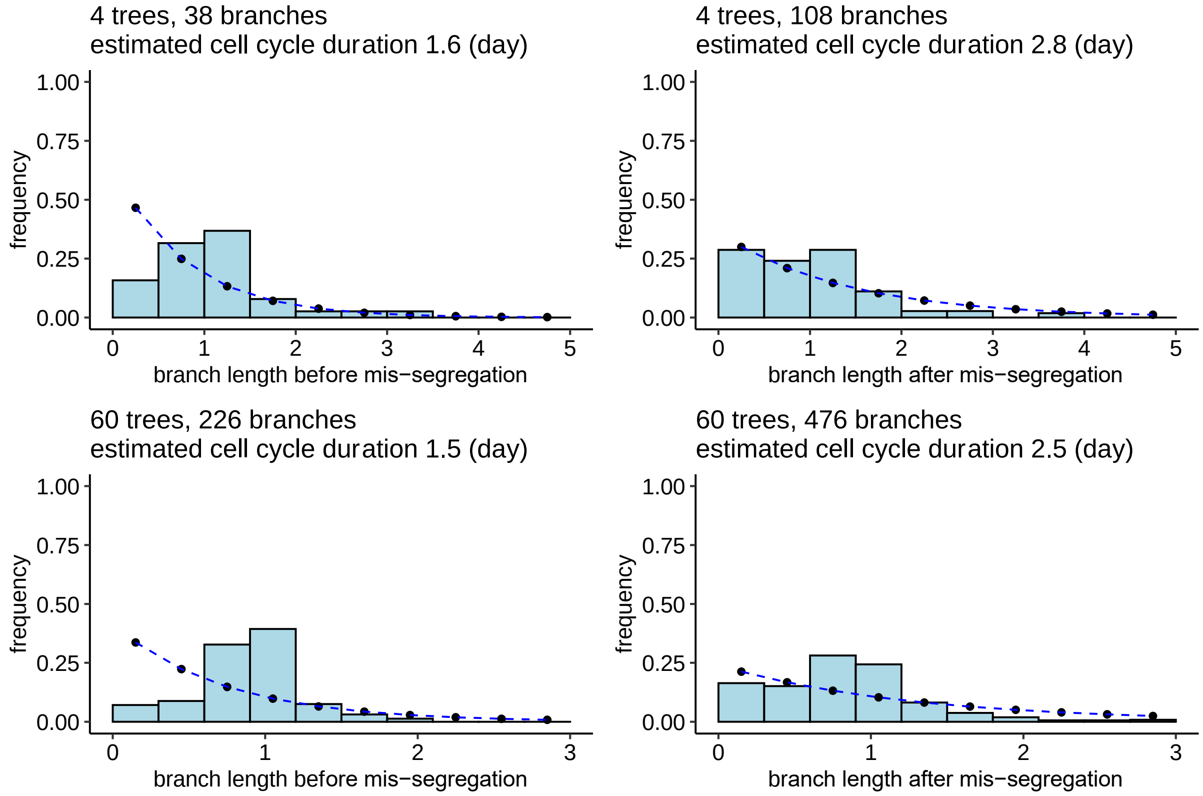

Supplementary Figure 5. Histogram of branch lengths in days (time between two consecutive cell divisions or time from the last division to the time when imaging stops) before and after mis-segregation in the real mitotic trees (Top 2 panels: dataset PDTO-9 #1,

#4, #5 and #7; Bottom 2 panels: 60 additional datasets). All the branches in the mitotic trees are included, except for eight basal branches in PDTO-9 #1, #4, #5 and #7. The estimated cell cycle duration time is the inverse of the maximum likelihood estimate of birth rate, which was computed based on joint likelihood of all the trees in consideration assuming a pure birth process. The blue dashed line shows the expected frequency of observed branch lengths based on estimated birth rate.

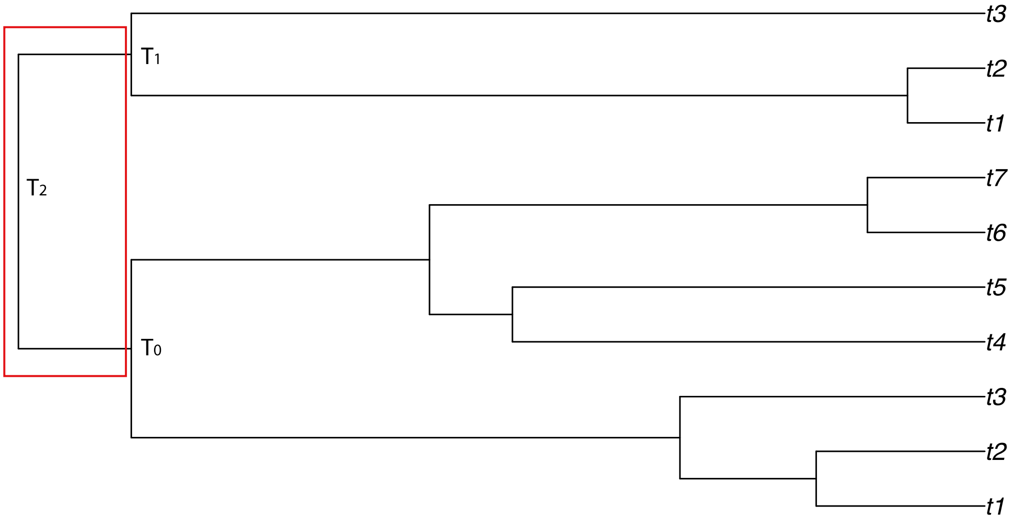

Supplementary Figure 6. The illustration of a simulated tree under H_1 with $b_0 = 0.65$, $b_1 = 0.4$, and $t = 3$. To get a tree with different birth rates, we simulated one subtree T_0 with b_0 and t, another subtree T_1 with b_1 and t, and a third subtree T_2 with b_0 and 2 tips. Then we merged them into a single tree by connecting the root of T_0 and T_1 to the two tips of T_2. To avoid bias, the branches of T_2 were excluded when doing likelihood-ratio test.

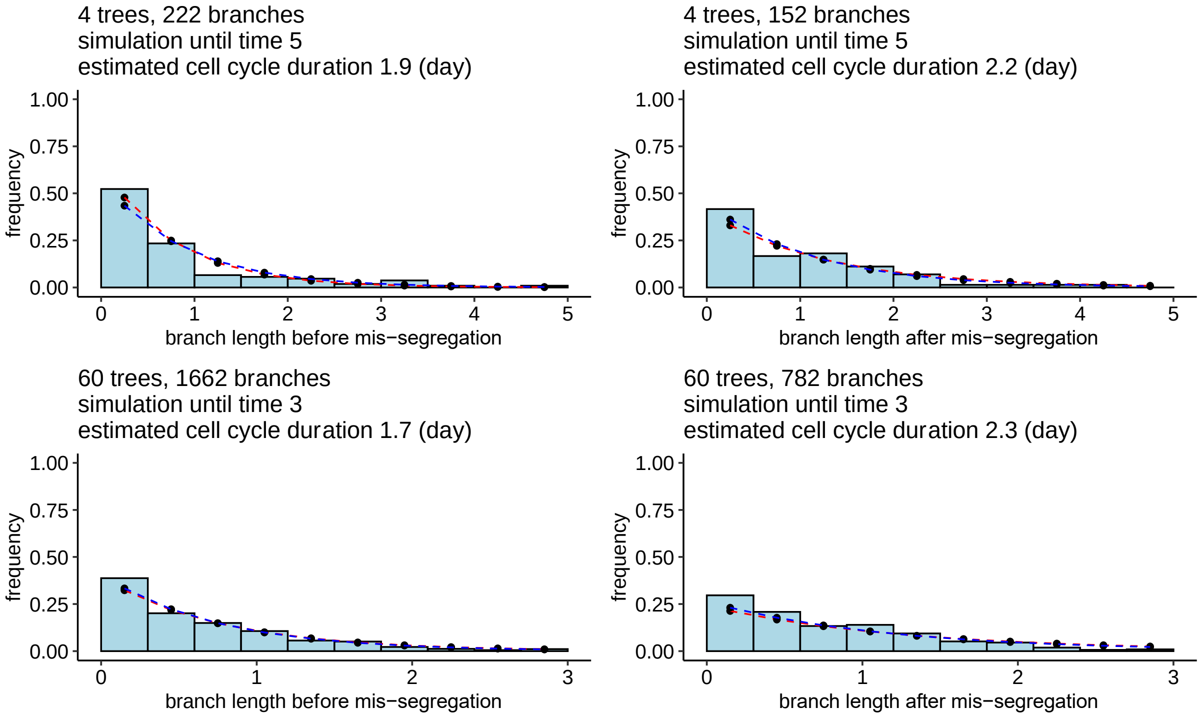

Supplementary Figure 7. Histogram of branch lengths before and after mis-segregation in trees simulated under H_1 with $b_0 = 0.65$ and $b_1 = 0.4$. We consider all the branches in the subtree simulated with b_0 (b_1) as before (after) mis-segregation. The blue (red) dashed line shows the expected frequency of observed branch lengths based on estimated (real) birth rate.

Supplementary Table 3. The power in detecting birth rate changes of 0.25 ($b_0 = 0.65$ and $b_1 = 0.4$) on simulated individual trees and joint trees of different sizes.

#joint trees	time	mean power
1	3	0.095
1	5	0.312
4	3	0.429
4	5	0.899
60	3	1.000
60	5	1.000

For the purpose of modelling the birth rates, we assumed pure birth and ignored death rate. We explored how the exclusion of death rate affected our results.

In the four-tree dataset, there are two dead cells, one in PDTO-9 #1 and one in PDTO-9 #7. In the 60-tree dataset, 18 of them have apoptotic events (1 with 3 dead cells, 4 with 2 dead cells and 13 with 1 dead cells). To get a simple estimate of the death rate, we simulated tree datasets of different sizes (4 and 60) with fixed birth rate 0.5 and a series of death rates (0.05, 0.04, 0.03, 0.02, and 0.01). To imitate real data, the four-tree datasets were simulated with $t = 5$ and the 60-tree datasets were simulated with $t = 3$. We then counted the number of trees with apoptotic events in each simulated dataset. We ran 100 replicates and computed the mean number of trees with apoptotic events. For the simulated four-tree datasets, there are on average 2 trees with apoptotic events when the death rate is 0.02. For the simulated 60-tree datasets, there are on average 20 trees with apoptotic events when the death rate is 0.03. Therefore, the death rate is about one order of magnitude lower than the birth rate. When death rate is not considered in the model, the birth rate may be slightly overestimated as the branches with dead cells are typically shorter. We applied LRT on 42 of 60 real trees without apoptotic events (p-value $5.24203e-05$), the estimations of birth rates ($b_0 = 0.69$, $b_1 = 0.40$) under H_1 are similar to those estimated when all the 60 trees were considered ($b_0 = 0.68$, $b_1 = 0.40$), suggesting that the assumption of pure birth does not affect the results or conclusions.

1 Williams, M. J. *et al.* Quantification of subclonal selection in cancer from bulk sequencing data. *Nat Genet* **50**, 895-903, doi:10.1038/s41588-018-0128-6 (2018).

2 Toni, T., Welch, D., Strelkowa, N., Ipsen, A. & Stumpf, M. P. Approximate Bayesian computation scheme for parameter inference and model selection in dynamical systems. *J R Soc Interface* **6**, 187-202, doi:10.1098/rsif.2008.0172 (2009).

3 François, O. & Laval, G. Deviance Information Criteria for Model Selection in Approximate Bayesian Computation. *Statistical Applications in Genetics and Molecular Biology* **10**, doi:10.2202/1544-6115.1678 (2011).

4 Nee, S. Inferring speciation rates from phylogenies. *Evolution* **55** (4), 661-668, doi:10.1111/j.0014-3820.2001.tb00801.x (2001).

5 Paradis E & Schliep K. ape 5.0: an environment for modern phylogenetics and evolutionary analyses in R. *Bioinformatics* **35**, 526-528, doi:10.1093/bioinformatics/bty633 (2019).
