# Supplementary material for: Reconstructing single-cell karyotype alterations in colorectal cancer identifies punctuated and gradual diversification patterns
Source: Nat Genet. 2021 Jul 1;53(8):1187–95. doi: 10.1038/s41588-021-00891-2 (PMC8346364; doi:10.1038/s41588-021-00891-2)
Supplement: Supplementary file 2 — Reporting Summary [file 41588_2021_891_MOESM2_ESM.pdf]

nature research

Corresponding author(s): Snippert, Hugo J.G.

Last updated by author(s): May 7th 2021

# Reporting Summary

Nature Research wishes to improve the reproducibility of the work that we publish. This form provides structure for consistency and transparency in reporting. For further information on Nature Research policies, see our Editorial Policies and the Editorial Policy Checklist.

## Statistics

For all statistical analyses, confirm that the following items are present in the figure legend, table legend, main text, or Methods section.

| n/a | Confirmed | |
|---|---|---|
| ☐ | ☒ | The exact sample size (*n*) for each experimental group/condition, given as a discrete number and unit of measurement |
| ☐ | ☒ | A statement on whether measurements were taken from distinct samples or whether the same sample was measured repeatedly |
| ☐ | ☒ | The statistical test(s) used AND whether they are one- or two-sided<br>*Only common tests should be described solely by name; describe more complex techniques in the Methods section.* |
| ☒ | ☐ | A description of all covariates tested |
| ☐ | ☒ | A description of any assumptions or corrections, such as tests of normality and adjustment for multiple comparisons |
| ☐ | ☒ | A full description of the statistical parameters including central tendency (e.g. means) or other basic estimates (e.g. regression coefficient) AND variation (e.g. standard deviation) or associated estimates of uncertainty (e.g. confidence intervals) |
| ☐ | ☒ | For null hypothesis testing, the test statistic (e.g. *F*, *t*, *r*) with confidence intervals, effect sizes, degrees of freedom and *P* value noted<br>*Give P values as exact values whenever suitable.* |
| ☒ | ☐ | For Bayesian analysis, information on the choice of priors and Markov chain Monte Carlo settings |
| ☒ | ☐ | For hierarchical and complex designs, identification of the appropriate level for tests and full reporting of outcomes |
| ☒ | ☐ | Estimates of effect sizes (e.g. Cohen's *d*, Pearson's *r*), indicating how they were calculated |

*Our web collection on statistics for biologists contains articles on many of the points above.*

## Software and code

Policy information about availability of computer code

| | |
|---|---|
| Data collection | The sequencing was carried out on an Illumina NextSeq500 and NovaSeq6000 using proprietary software from Illumina preloaded on the machine.<br>Live-cell imaging was performed on an SP8X Leica laser scanning confocal microscope, equipped with Argon laser and white-light laser, using proprietary software preloaded on the machine for acquisition (Leica LAS X), as well as a spinning-disc confocal microscope (Nikon) with preloaded proprietary software (NIS-Elements). Zeiss LSM510 was used to acquire 3D image of fresh organoid/tumor samples prior single-cell picking. |
| Data analysis | Full pipeline description and settings for alignment of sequence reads to human genome reference and variant calling are available at: https://github.com/UMCUGenetics/IAP. Manta v0.29.5 was used with standard settings to detect structural variants.<br>The copy number status of each single cell was analyzed using Ginkgo; pipeline available at: https://github.com/robertaboukhalil/ginkgo.<br>BAM files (which contain sequence alignment data) were converted to BED format files using BEDtools v2.25.0. Copy number profiles are presented in heatmaps which were generated using R (v3.4.3) package ggplot2. R package karyoploteR was used to generate a genome-wide map of all de novo CNA events in PDTO-9 and -19b.<br>Image analysis was performed using Leica LAS X and NIS-Elements proprietary software preloaded on the microscopes, Imaris Software (version 9.2) for 3D rendering and with the freely available Imagej/Fiji (version 1.51n) to analyze spinning disc data.<br>Microsoft excel (version 2019). |

For manuscripts utilizing custom algorithms or software that are central to the research but not yet described in published literature, software must be made available to editors and reviewers. We strongly encourage code deposition in a community repository (e.g. GitHub). See the Nature Research guidelines for submitting code & software for further information.

## Data

Policy information about availability of data

All manuscripts must include a data availability statement. This statement should provide the following information, where applicable:

- Accession codes, unique identifiers, or web links for publicly available datasets
- A list of figures that have associated raw data
- A description of any restrictions on data availability

The raw sequencing datasets (BAM files) generated and analyzed during the current study are available in the European Genome-phenome Archive (EGA, hosted by the EBI and CRG) repository with study ID EGAS00001003812.
The copy-number segment calls of the sequencing data of all single cells in this study are publicly available on Zenodo (OpenAIRE project, commissioned by the European Commission) with study number DOI: 10.5281/zenodo.4732372.

# Field-specific reporting

Please select the one below that is the best fit for your research. If you are not sure, read the appropriate sections before making your selection.

☒ Life sciences ☐ Behavioural & social sciences ☐ Ecological, evolutionary & environmental sciences

For a reference copy of the document with all sections, see nature.com/documents/nr-reporting-summary-flat.pdf

# Life sciences study design

All studies must disclose on these points even when the disclosure is negative.

| | |
|---|---|
| Sample size | No pre-study calculations were carried out to determine sample size. We sequenced the maximum amount of single cells derived from 12 distinct clonal colorectal cancer organoids. In addition, we included an additional 66 photoconverted single cells (all derived from individual clonal organoids) with their corresponding bulk sample. Furthermore, we sequenced a high fraction of single cells from clonal tumor fragments isolated from two individual colorectal cancer glands and from a healthy crypt. Results obtained were highly significant and consistent and did not require additional experiments. Sample sizes are indicated throughout manuscript and in accompanying data files. |
| Data exclusions | Throughout the study of this manuscript we excluded single cell sequencing data with insufficient mapped reads (< 80%). Single cells with excessive amount of noise within copy number profile were excluded, generally when having >2 the standard deviation of copy number ratios as compared to the average standard deviation of the mean of the total data set. For the whole study, the total number of cells that were excluded based on above criteria were 45 cells (6%). Number of cells excluded per dataset are indicated in Supplementary table 2. |
| Replication | The main findings that chromosomal copy-number alterations of varying complexity can arise within a few cell generations, and that sub-chromosomal fragments lacking centromeric regions are prone to replication and collective missegregation during consecutive cell divisions are detected in multiple clonal organoids.<br>We restricted our study to reciprocal gains and losses between sister cells, non-reciprocal whole-chromosome gains or losses, and non reciprocal sub-chromosomal CNAs represented in more than one cell thereby significantly improving our confidence of focusing on true de novo copy-number variations.<br>We confirmed that ongoing generation of gradual and punctuated karyotype diversity observed in PDTOs can occur to a similar degree in vivo. Each single-cell sequencing effort of clonal organoids or tumor structures, is described, presented and evaluated separately in the manuscript. In addition, when experiments were replicated and data summarized, the number of times are indicated in each figure legend. All attempts at replication were successful and have been included in the datasets. |
| Randomization | Individual organoids to be analyzed with single-cell DNA sequencing were chosen randomly (per definition is genomic diversification patterns not known at the moment of organoid selection). For experiments where multi-organoid data is summarized, all organoids within a field of view were analyzed to minimize sampling bias (~10-20 organoids/field of view, 4 positions). |
| Blinding | Data acquisition was not performed blindly, due to PDO line specific phenotypes and/or characteristics. |

# Reporting for specific materials, systems and methods

We require information from authors about some types of materials, experimental systems and methods used in many studies. Here, indicate whether each material, system or method listed is relevant to your study. If you are not sure if a list item applies to your research, read the appropriate section before selecting a response.

## Materials & experimental systems

| n/a | Involved in the study |
|---|---|
| ☒ | ☐ Antibodies |
| ☐ | ☒ Eukaryotic cell lines |
| ☒ | ☐ Palaeontology and archaeology |
| ☒ | ☐ Animals and other organisms |
| ☐ | ☒ Human research participants |
| ☒ | ☐ Clinical data |
| ☒ | ☐ Dual use research of concern |

## Methods

| n/a | Involved in the study |
|---|---|
| ☒ | ☐ ChIP-seq |
| ☒ | ☐ Flow cytometry |
| ☒ | ☐ MRI-based neuroimaging |

# Eukaryotic cell lines

Policy information about cell lines

| | |
|---|---|
| Cell line source(s) | Organoid lines derived of human colorectal cancer tissues were previsouly described by Van de Wetering et al. 2015 Cell. |
| Authentication | Multiple point mutations that are unique per patient organoid sample were repeatedly identified and confirmed. |
| Mycoplasma contamination | Samples have repeatedly been tested negative for Mycoplasma. |
| Commonly misidentified lines (See ICLAC register) | Not applicable |

# Human research participants

Policy information about studies involving human research participants

| | |
|---|---|
| Population characteristics | Fresh-frozen human colorectal cancer tissue was obtained from patients from the UCL Hospitals. All surgical resected samples were collected from patients who had given informed consent. Frozen subsamples of random individuals have been sent to the Snippert laboratory (treatment, age and gender not disclosed), where individual glands (cancer and adjacent normal) were subsequently isolated upon thawing of the sample for prospective single-cell picking and genome analysis. |
| Recruitment | Available tissue from patients with informed consent were collected without any bias in selection of the participants. |
| Ethics oversight | Fresh-frozen tissue samples were collected from UCL Hospitals under ethical approval 11/LO/1613 and via the UCLH Biobank (15/YH/0311) |

Note that full information on the approval of the study protocol must also be provided in the manuscript.
